# 8-Hydroxydaidzein Induces Apoptosis and Inhibits AML-Associated Gene Expression in U-937 Cells: Potential Phytochemical for AML Treatment

**DOI:** 10.3390/biom13111575

**Published:** 2023-10-26

**Authors:** Pei-Shan Wu, Chih-Yang Wang, Hao-Jen Hsu, Jui-Hung Yen, Ming-Jiuan Wu

**Affiliations:** 1 Department of Pharmacy, Chia Nan University of Pharmacy and Science, Tainan 717301, Taiwan; dc7575@gmail.com; 2 Department of Biotechnology, Chia Nan University of Pharmacy and Science, Tainan 717301, Taiwan; 3 Ph.D. Program for Cancer Molecular Biology and Drug Discovery, Taipei Medical University, Taipei 110301, Taiwan; chihyang@tmu.edu.tw; 4 Graduate Institute of Cancer Biology and Drug Discovery, Taipei Medical University, Taipei 110301, Taiwan; 5 Department of Biomedical Sciences and Engineering, Tzu Chi University, Hualien 970, Taiwan; hjhsu32@mail.tcu.edu.tw; 6 Department of Molecular Biology and Human Genetics, Tzu Chi University, Hualien 970374, Taiwan; imyenjh@mail.tcu.edu.tw; 7 Institute of Medical Sciences, Tzu Chi University, Hualien 970374, Taiwan

**Keywords:** 8-hydroxydaidzein, apoptosis, CDK6, caspase-7, acute myeloid leukemia

## Abstract

Background: 8-hydroxydaidzein (8-OHD) is a compound derived from daidzein, known for its anti-inflammatory and anti-proliferative properties in K562 human chronic myeloid leukemia (CML) cells. However, its effects on acute myeloid leukemia (AML) cells have not been fully understood. Method: To investigate its potential anti-AML mechanism, we employed an integrated in vitro–in silico approach. Results: Our findings demonstrate that 8-OHD suppresses the expression of CDK6 and CCND2 proteins and induces cell apoptosis in U-937 cells by activating Caspase-7 and cleaving PARP-1. Microarray analysis revealed that 8-OHD downregulates differentially expressed genes (DEGs) associated with rRNA processing and ribosome biogenesis pathways. Moreover, AML-target genes, including *CCND2*, *MYC*, *NPM1*, *FLT3,* and *TERT*, were downregulated by 8-OHD. Additionally, molecular docking software predicted that 8-OHD has the potential to interact with CDK6, FLT3, and TERT proteins, thereby reducing their activity and inhibiting cell proliferation. Notably, we discovered a synergic pharmacological interaction between 8-OHD and cytarabine (Ara-C). Conclusions: Overall, this study provides insights into the therapeutic applications of 8-OHD in treating AML and elucidates its underlying mechanisms of action.

## 1. Introduction

Isoflavones, known as dietary phytoestrogens, are primarily synthesized by the Fabaceae family [1]. These phytochemical agents are used in alternative therapies for cancers, metabolic syndrome, osteoporosis, and postmenopausal symptoms [2,3,4,5]. 8-Hydroxydaidzein (also known as 8-OHD, 7,8,4’-trihydroxyisoflavone, and NSC-678112) is one of the metabolites of daidzein found in the human body [6]. Recent research indicated its potential therapeutic effects for inflammation, carcinogenesis, melanogenesis, and neuroprotection [7,8,9,10,11,12,13]. 8-OHD also has strong anti-proliferative activity in human promyelocytic leukemia HL-60 cells and K562 CML cells [14,15,16].

Leukemia is a hematological disorder that can be classified based on the type of cells involved and the rate of disease progression. Acute myeloid leukemia (AML), also known as acute myelocytic, myelogenous, or granulocytic leukemia, as well as acute non-lymphocytic leukemia, is the most prevalent type of aggressive leukemia in adults [17]. AML is a complex disease that encompasses several subtypes, each presenting with a diverse array of genetic abnormalities and variable prognoses [18]. With the progress of next-generation sequencing (NGS), driver mutations in AML have been reported [19]. These include Nucleophosmin 1 (NPM1), FMS-related tyrosine kinase 3 (FLT3), transcription factor RUNX1, epigenetic modifier DNA methyltransferase (DNMT)3A and others [19].

Ribosome biogenesis, the production of new ribosomes, is a complex process that involves a multitude of factors and is highly regulated. Dysregulation of ribosome biogenesis is a hallmark of cancer and has been implicated in the development and progression of several cancer types. Therefore, targeting ribosome biogenesis has emerged as a promising strategy for cancer therapy. Recent understanding of the molecular mechanisms of ribosome biogenesis has led to the development of ribosome biogenesis inhibitors that selectively target various steps of this process and possess fewer genotoxic side effects than conventional therapies [20,21,22].

Despite extensive research to understand the pathogenesis of AML, the standard therapy, a combination of anthracycline and cytarabine (Ara-C), has remained unchanged for several decades. The overall survival rates for patients with AML remain poor, particularly for older patients or those with relapsed or refractory disease [23]. There is an urgent need to discover and develop new drugs for AML that offer improved efficacy, reduced toxicity, and potential for combination therapy with existing drugs in order to enhance patient outcomes. Phytochemicals have been documented to influence the treatment of AML through conjugation therapy in various ways. These include altering chromatin structure by modulating the activities of DNMTs or histone deacetylases (HDACs), changing the expression and activities of specific DNA repair proteins, and inducing apoptotic effects through modulating signaling pathways [24].

In this study, we aimed to investigate the effects and underlying mechanisms of 8-OHD on AML cells. An integrated in vitro–in silico approach was employed. The cytotoxic effects of 8-OHD on three distinct AML cell lines were first investigated. A microarray analysis was then utilized to investigate differentially expressed genes (DEGs) in U-937 cells, a model cell line derived from an adult AML patient, that were treated with 8-OHD. FEA (Functional enrichment analysis) was performed to see if the DEGs appeared in particular gene ontology (GO) and Kyoto Encyclopedia of Genes and Genomes (KEGG) pathways [25,26]. Gene set enrichment analysis (GSEA) was then employed to verify whether 8-OHD treatment activated/inhibited the functional pathways [27]. Furthermore, AML target genes were obtained from the DisGeNET database [28], and the overlapping part of DEGs and AML targets are the potential 8-OHD target AML genes. A docking simulation was then performed to investigate plausible protein targets of 8-OHD. Finally, the combined cytotoxicity of Ara-C and 8-OHD in U-937 cells was also analyzed.

## 2. Materials and Methods

### 2.1. Preparation of 8-Hydroxydaidzein (8-OHD, 7,8,4ʹ-Trihydoxyisoflavone, NSC 678112)

**Figure 1:**
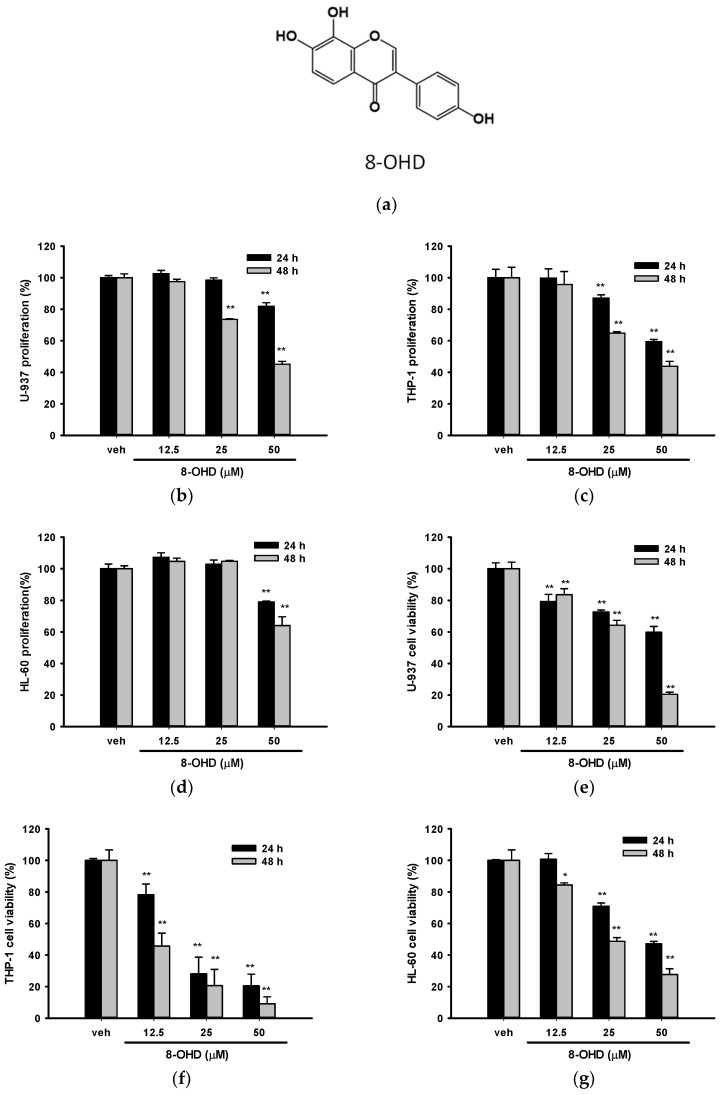
Effects of 8-OHD on the cell proliferation and cell viability of U-937, THP-1, and HL-60 cells. (**a**) Chemical structure of 8-OHD. (**b**–**d**) U-937, THP-1, and HL-60 cells were treated with vehicle (0.1% DMSO) or 8-OHD (12.5–50 μM) for the indicated period, and cell proliferation was measured using the MTT assay. (**e**–**g**) Cell viability was examined using the trypan blue exclusion test. The experiments were repeated three times. These data represent the mean ± SD of three independent experiments. * *p* < 0.05 and ** *p* < 0.01 represent significant differences compared with the vehicle-treated cells.

8-OHD was isolated from soybean fermented by *Aspergillus oryzae*, and the NMR spectral data and purification of 8-OHD were previously reported [8,9,12]. The chemical structure of 8-OHD is shown in Figure 1a.

### 2.2. Cell Culture

The U-937 cell line was derived from an adult with AML in a metastatic state, THP-1 was derived from a child with AML, while HL-60 was derived from an adult with APL, a subtype of AML. The U-937, THP-1, and HL-60 cells were provided by the Bioresource Collection and Research Center (Hsinchu, Taiwan). The U-937 and HL-60 cells were cultured in RPMI-1640 Hybri-Max medium (Sigma-Aldrich Co., St. Louis, MO, USA) supplemented with 10% fetal bovine serum (FBS), 1% nonessential amino acids (NEAA), 100 units/mL of penicillin, and 100 µg/mL of streptomycin (Thermo Fisher Scientific, Inc., Rockford, IL, USA). The THP-1 cells were cultured in the same medium as above but supplemented with an additional 1 mM sodium pyruvate and 0.05 mM 2-mercaptoethanol (Thermo Fisher Scientific). Cells were cultured in a 5% CO_2_ incubator at 37 °C and passaged twice a week to maintain a cell density between 2 × 10^5^ and 10^6^/mL.

### 2.3. Cell Proliferation and Viability Analysis

All three cell lines (5 × 10^5^/mL) were treated with vehicle (0.1% DMSO) or 8-OHD (12.5–50 μM) for 24 h or 48 h. Cell proliferation was then analyzed by adding 1/10 volume of 0.5% MTT (in PBS) and incubating for 3 h. After incubation, the MTT solution was removed by centrifugation, and the formazan crystals were dissolved in DMSO. The absorbance of the resulting solution was measured at 550 nm [29].

The viability of cells following treatment was assessed using the trypan blue exclusion test, as previously described in the literature [29]. The concentration (µM) causing 50% cell death (IC_50_) was determined using an online four-parameter logistic (4PL) curve calculator (AAT Bioquest, Inc., Sunnyvale, CA, USA).

### 2.4. Apoptosis Analysis

U-937 cells were treated with 8-OHD for 24 and 48 h and then subjected to Annexin V-FITC apoptosis detection kit (Strong Biotech Crop, Taipei, Taiwan). The stained cells were examined using inverted fluorescence microscopy (ECLIPSE Ti-E, Nikon Instruments Inc., Minato, Tokyo, Japan) according to manufacturer’s manual.

### 2.5. Western Blot Analysis

Following treatment, U-937 cells were lysed using RIPA lysis buffer to prepare cell lysates, while nuclear extracts were prepared using a nuclear extraction kit (Cayman, Ann Arbor, MI, USA). The protein concentration was quantified using the Bradford binding assay (Bio-Rad Laboratories, Hercules, CA, USA), and bovine serum albumin (BSA) was used as a standard.

Equal amounts of protein were separated on SDS-PAGE (8–10%) and then transferred to polyvinylidene difluoride (PVDF) membranes (Hybond-P, GE Healthcare, Chicago, IL, USA) in CAPS buffer (10 mM, pH 10.5, 10% methanol) at 20 volts overnight at 4 °C. The membranes were blocked in freshly made blocking buffer (5% skim milk in PBS with 0.05% Tween 20, pH 7.4) for 8 h at room temperature and then probed with specific primary antibody (Table 1) overnight at 4 °C, followed by horseradish peroxidase (HRP)-conjugated secondary antibody (Jackson ImmunoResearch, West Grove, PA, USA) for 1 h at room temperature. The protein of interest was detected by enhanced chemiluminescence detection (GE Healthcare).

### 2.6. RNA Extraction and Reverse Transcription Real-Time PCR (RT-qPCR)

After 24 h treatment, total RNA was extracted from U-937 cells with the Illustra RNA Spin Mini RNA Isolation Kit (GE Healthcare). The High-Capacity cDNA Archive kit (Thermo Fisher Scientific) was used for reverse transcription. The resulting cDNA was utilized for qPCR using a Power SYBR Green PCR Master Mix (Thermo Fisher Scientific) with the appropriate primer pair (Table 2) at 95 °C for 2 min, 40 cycles at 94 °C for 15 s, and 60 °C for 60 s (ABI StepOne Real-Time PCR System). The relative mRNA expression was normalized with *GAPDH* expression and then calculated by the 2^−ΔΔCT^ method. Specificity verification was performed by melting curve.

### 2.7. Microarray Analysis

U-937 cells were treated with vehicle (0.1% DMSO) or 8-OHD (50 μM) for 24 h. Microarray analysis was performed using the Human OneArray Plus platform (Phalanx Biotech, Hsinchu, Taiwan) with array version HOA 7.1 as previously described [30,31]. The fluorescence intensities of each spot were analyzed and processed by GenePix 4.1 software (Molecular Devices, Sunnyvale, CA, USA) and Rosetta Resolver System (Rosetta Biosoftware, Seattle, WA, USA), respectively. Differentially expressed genes (DEGs) related to the treatment of 8-OHD were screened, and those with log_2_ (fold change value) larger than 1.0 or less than −1.0 and *p* < 0.05 were selected.

### 2.8. Analysis of Gene Ontology (GO) and Kyoto Encyclopedia of Genes and Genomes (KEGG) Pathways, as Well as Gene Set Enrichment Analysis (GSEA)

To assess the functions and significantly enriched pathways of the DEGs, GO term and KEGG pathways were further analyzed using the Database for Annotation, Visualization, and Integrated Discovery (DAVID 2021, http://david.ncifcrf.gov, accessed on 3 May 2022), an online biological information database and *p* < 0.05 was used as the cut-off criterion [32,33,34]. Molecular functions and the pathway database from the MetaCore platform (GeneGo, St. Joseph, MI, USA, accessed on 25 May, 2022) were further used to explore potential signaling pathways modulated by 8-OHD. Gene Set Enrichment Analysis (GSEA) [27] (v.4.3.2, http://www.gsea-msigdb.org/gsea/index.jsp, accessed on 25 March 2023) was further used to verify the statistically significant molecular pathway with 8-OHD treatment.

### 2.9. Analysis of AML-Target DEGs

DisGeNET (https://www.disgenet.org/search, accessed on 16 August, 2022) is a comprehensive online platform integrating information on human disease-associated genes and variants [28]. AML-related human genes were retrieved from DisGeNET, and two entries were found: AML-M1, CUI: C0026998 and AML-M2, CUI: C1879321. A Venn diagram of gene intersection between the three gene sets of 8-OHD-downregulated DEGs, C0026998, and C1879321 was plotted and visualized with Venny 2.1.0 (https://csbg.cnb.csic.es/BioinfoGP/venny.html, accessed on 30 August, 2022). Overlapping genes among the downregulated DEGs and the AML-related genes were defined as 8-OHD target AML genes.

### 2.10. Prediction of Protein Targets

Swiss Target Prediction is a web server that predicts the targets of bioactive molecules by reverse screening. The predictions are performed by searching for similar molecules, in 2D and 3D, within 376,342 compounds known to be experimentally active on 3068 protein targets [35]. By importing the SMILE structure of 8-OHD into Swiss Target Prediction database, all potential target proteins can be found.

### 2.11. Molecular Docking of 8-OHD to Possible Target Proteins

The Molecular Operating Environment software (MOE2020.09) was employed to predict the binding modes of 8-OHD to various target proteins, FLT3, CDK6, and TERT, which were obtained from Swiss Target Prediction. The DOCK module with “Induced fit” refinement was applied to improve the accuracy of the predictions. Prior to docking, 8-OHD was manually built in the MOE software (MOE2019.01) package and docked with the binding domain of different proteins (PDB codes: 6JQR for FLT3; 4AUA for CDK6; 6USR for TERT). The MOE software was also used to remove crystal water molecules and add missing short loops, and the potential energy was minimized. A force field-based scoring function, GBVI/WSA dG, was employed to estimate the binding free energy of the ligand to protein [36]. The preferable binding sites for 8-OHD were determined by identifying the lowest binding free energy and the lowest S value of the scoring function.

### 2.12. Analysis of Drug Combination Effects

We utilized the Combenefit 2.021 software [37] to quantify the combined cytotoxicity effect of 8-OHD and Ara-C in U-937 cells. Within Combenefit, we employed the Loewe and Highest Single Agent (HAS) models to quantify the divergence between the anticipated additive effect and the actual effect of the drug combination. When the observed effect surpasses the additive effect, the synergy score is positive; conversely, it is negative if the observed effect falls short. A higher synergy score indicates a more pronounced synergy within the drug combination [38].

### 2.13. Statistical Analysis

All experiments were repeated at least three times, and the values were expressed as the mean ± SD. The results were analyzed using One-way ANOVA with Dunnett’s post hoc test, and a *p* value < 0.05 was considered statistically significant.

## 3. Results and Discussion

### 3.1. 8-OHD Reduces the Proliferation and Cell Viability in AML Cells

We first examined the effect of 8-OHD on cell proliferation by MTT assay and cell viability by trypan blue exclusion analysis for these three cell lines. The concentrations that were tested had previously been examined in PBMC, and no cytotoxic effects were observed. Figure 1b–g show that 8-OHD (12.5–50 μM) significantly decreased cell proliferation and cell viability in a dose- and time-dependent manner in these three cell lines. In fact, trypan blue exclusion seems to be a better analytic method because antioxidant phytochemicals are likely to interfere with the MTT reduction assay [39]. We found that THP-1 was the most vulnerable to 8-OHD treatment, particularly after 48 h (Figure 1f).

The estimated concentrations that cause 50% cell death (IC_50_) at 24 and 48 h by a four-parameter logistic regression model for these three cell lines are summarized in Table 3. Because AML usually occurs in older adults [40], we thus chose U-937 cells for the following experiments.

### 3.2. 8-OHD Induces Apoptosis in U-937 Cells

**Figure 2:**
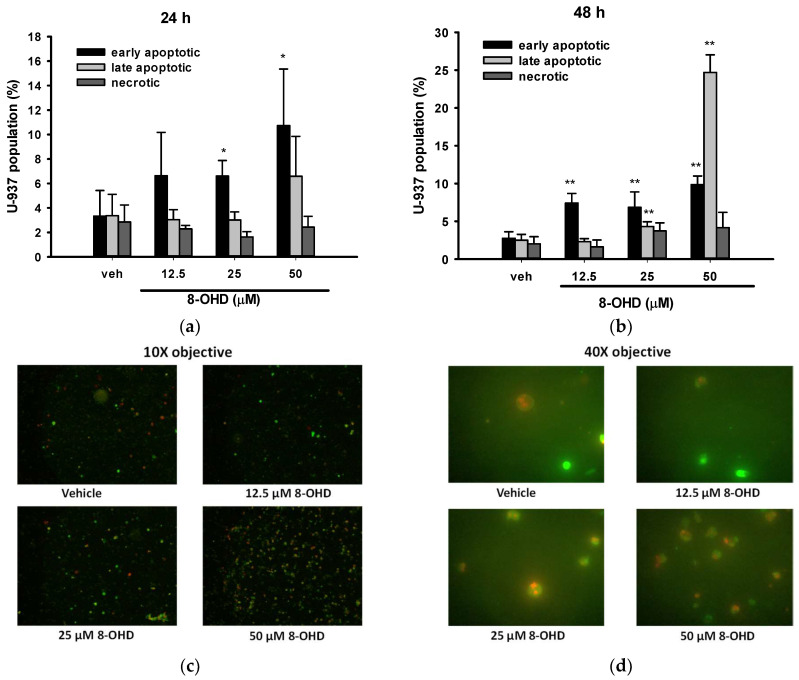
Quantitation of early apoptosis, late apoptosis, and necrosis by Annexin V and propidium iodide (PI) stain. U-937 cells were treated with various concentrations of 8-OHD for 24 or 48 h and stained with Annexin V-PI. Images were captured using ×10 and ×40 objectives, and apoptotic cells were identified by direct visualization under a fluorescent microscope. Cells stained for Annexin V are green, cells stained for PI are red, and cells stained for both are yellow under ×10 objective. Annexin V-positive and PI-negative cells are classified as early apoptotic, PI-positive and annexin V-negative cells are denoted as necrotic, and both Annexin V- and PI-positive are symbolized as late apoptotic in a population. (**a**,**b**) Quantitation of early apoptosis, late apoptosis, and necrosis after 24 and 48 h treatment with 8-OHD in U-937 cells. These data represent the mean ± SD of five different fields. * *p* < 0.05 and ** *p* < 0.01 represent significant differences compared with the vehicle-treated cells. (**c**,**d**) Representative merged images of U-937 cells treated with various levels of 8-OHD for 48 h under 10× and 40× objectives.

We proceeded with our investigation of cell death type induced by 8-OHD in U-937 cells. To do so, we utilized Annexin V-FITC/PI double staining and visualized the samples through fluorescence microscopy. Annexin V-FITC^+^/PI^−^ cells are classified as early apoptotic, Annexin V-FITC^−^/PI^+^ cells are denoted as necrotic, and both Annexin V-FITC^+^/PI^+^ are symbolized as late apoptotic in a population [41]. Five different fields of microscopy for each treatment were counted and summarized. We found that 8-OHD (25 and 50 μM) significantly induced early apoptosis after 24 h treatment (Figure 2a), while longer treatment (48 h) caused both early and late apoptosis as compared with vehicle (Figure 2b). No significant necrotic death could be found (Figure 2a,b). Figure 2c,d show the representative microscopy images of U-937 cells treated with different doses of 8-OHD for 48 h.

**Figure 3:**
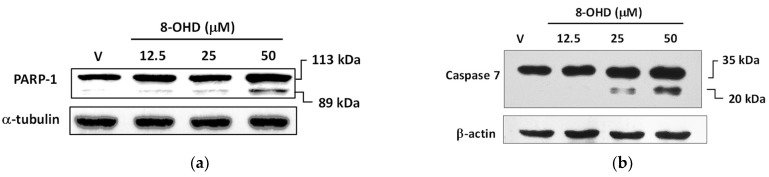
8-OHD causes poly (ADP-ribose) polymerase-1 (PARP-1) cleavage (**a**) and caspase-7 activation (**b**) in U-937 cells dose dependently. Total cell lysates were prepared 24 h after 8-OHD treatment, as described in Materials and Methods.

It is well known that poly (ADP-ribose) polymerase-1 (PARP-1) cleavage functions as a molecular switch between apoptotic and necrotic modes of death receptor-induced cell death [42]. Consistent with the data mentioned earlier, Figure 3a illustrates that the cleavage of PARP-1 was induced in a dose-dependent manner by 8-OHD (12.5–50 μM) treatment 24 h after administration. Notably, a dose of 50 μM, which is approximately 0.6 times the IC_50_, demonstrated significant effectiveness.

The crucial role of caspase-mediated proteolytic inactivation of PARP-1 in facilitating the successful completion of the apoptotic process is well-documented [43], with caspase-7 exhibiting the highest efficiency in cleaving PARP-1 [44]. Figure 3b demonstrates that 8-OHD dose dependly induced caspase-7 expression and cleavage.

### 3.3. Analysis of 8-OHD-Modulated Gene Expression, GO Term, and Pathway Enrichment

**Figure 4:**
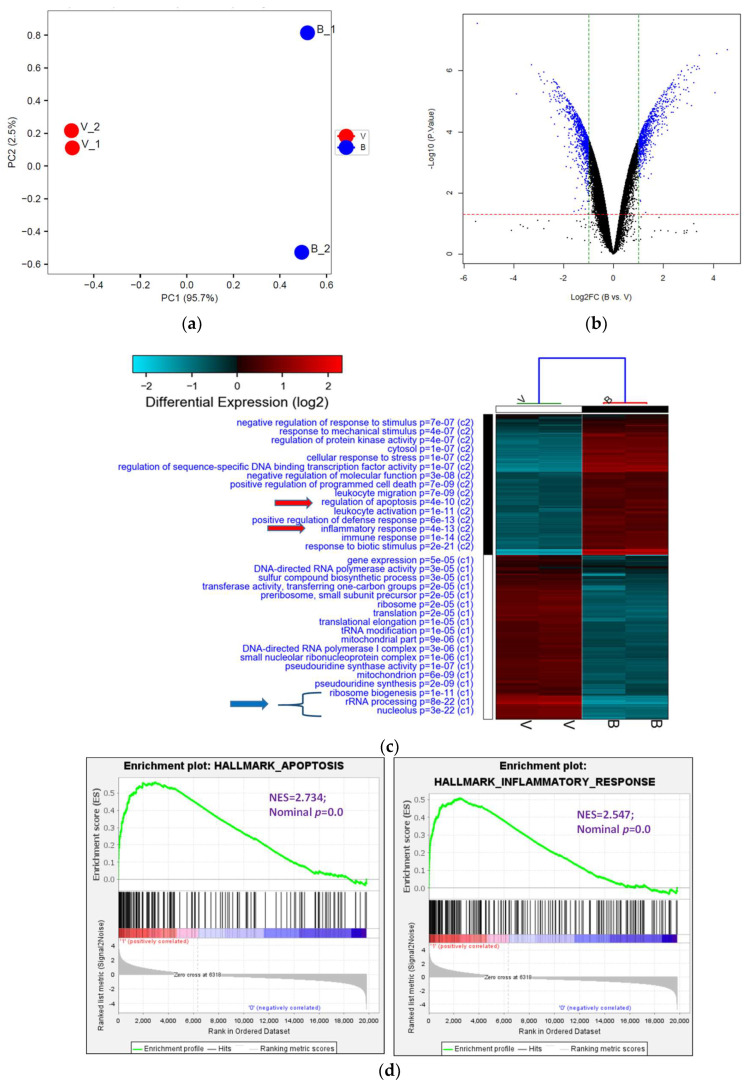

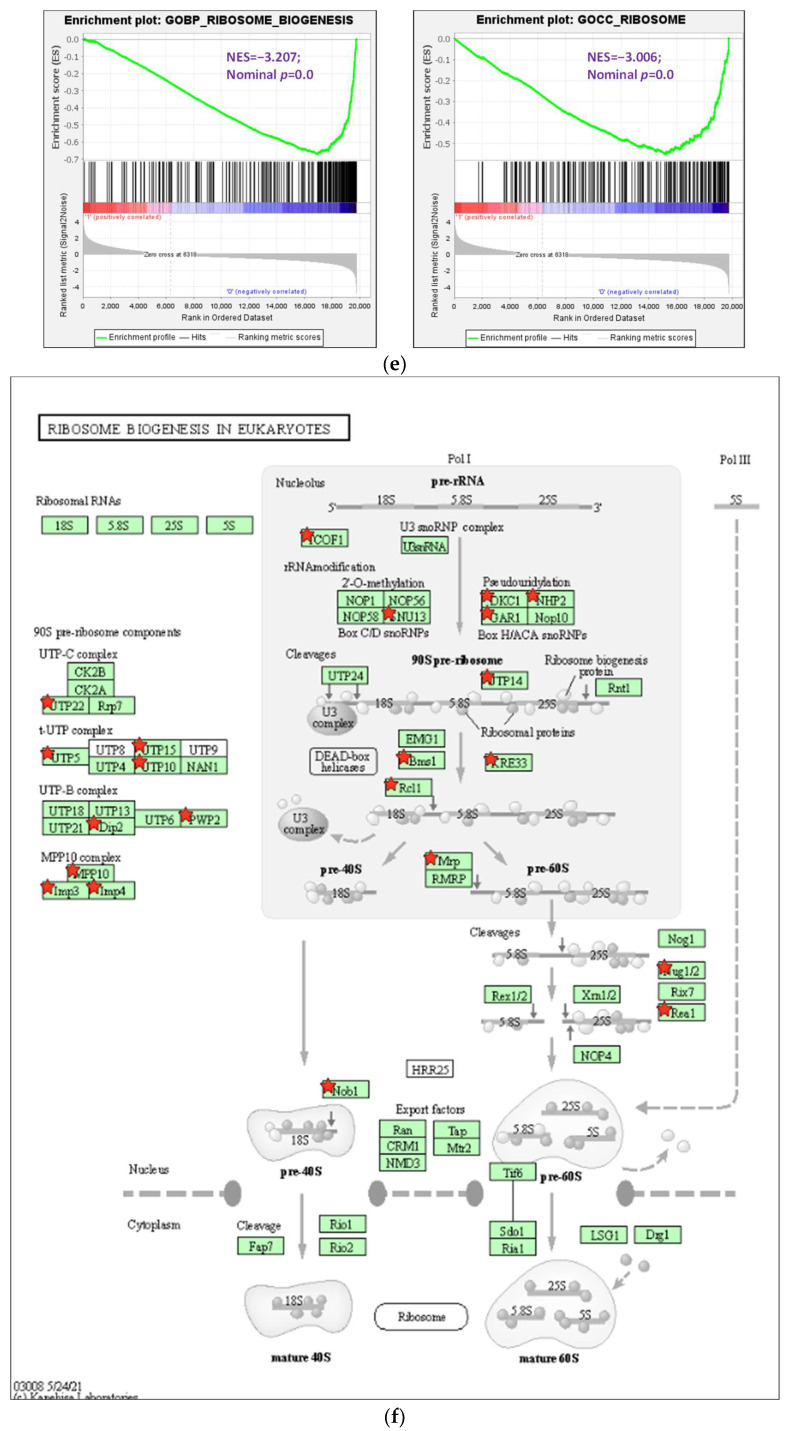

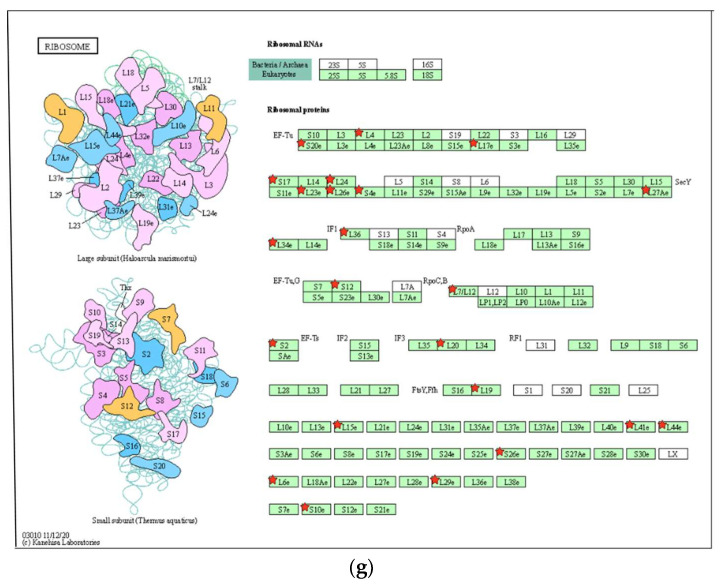
Analysis of Gene Ontology (GO) terms and Kyoto Encyclopedia of Genes and Genomes (KEGG) pathways of DEGs. (**a**) PCA plot of variants of two duplicated vehicles (V) and 50 μM 8-OHD (B). (**b**) The volcano plot of 50 μM 8-OHD (B) versus vehicle (V). Standard selection criteria to identify differentially expressed genes (DEGs) are established at log_2_ |fold change| > 1 and *p* < 0.05 (Blue dots in figure). (**c**) Cluster analysis of DEGs. GO terms listed in the upper half are terms of upregulated DEGs, while lower half are terms of downregulated DEGs. Only some of the representative GO terms are shown. (**d**) Gene Set Enrichment Analysis (GSEA) demonstrates that the signature “Hallmark apoptosis” and “Hallmark inflammatory response” gene sets are enriched with 8-OHD-upregulated genes. (**e**) GSEA demonstrates that “GOBP ribosome biogenesis” and “GOCC ribosome” are enriched with 8-OHD-downregulated genes. The barcode plot indicates the position of the genes in each gene set. The horizontal bar in graded color from red to blue indicates up- and down-regulated by 8-OHD. The vertical axis in the lower plot indicates the Ranked List Metric. (**f**) Kegg pathway—ribosome biogenesis in eukaryotes (hsa03008) (**g**) Kegg pathway—ribosome (hsa03010). The downregulated DEGs were marked with red stars.

The analysis of transcriptome provides an excellent approach to examining the alteration in RNA expression induced by stress. Therefore, it represents a valuable resource for making critical decisions in the early stages of drug discovery [45]. We, thus, investigated gene expression in 8-OHD-treated U-937 cells by human genome-wide microarray analysis. U-937 cells were treated with vehicle (0.1% DMSO) and 8-OHD (50 μM) for 24 h, and transcriptome profiles were analyzed using the Human OneArray system, which contains 25,765 known genes. Principal Component Analysis (PCA) was performed to evaluate differences between treatments. It was found that expression profiles of vehicle-treated (V) and 8-OHD-treated (B) were significantly different, and principal component 1 (PC1) could explain 95.7% variation between them (Figure 4a). This indicates that 50 μM 8-OHD treatment caused significant changes in gene expression in U-937 cells. Figure 4b shows that treatment of U-937 cells with 50 μM 8-OHD for 24 h yielded 676 upregulated differentially expressed genes (DEGs) (log_2_ (Fold change) > 1 and *p* < 0.05) and 748 downregulated DEGs (log_2_ (Fold change) < −1 and *p* < 0.05).

DEGs were then subjected to GO term enrichment analyses. The analysis revealed that 64 GO terms (C1) were enriched with downregulated DEGs, while 125 GO terms (C2) were enriched with upregulated DEGs (*p* < 0.05). The heatmap of selected enriched GO terms is shown in Figure 4c, and the complete list is provided in Supplemental Tables S1 and S2 (adjusted *p* < 0.05). It was found that 8-OHD-downregulated DEGs are strongly associated with rRNA processing and ribosome biogenesis. On the other hand, 8-OHD-upregulated DEGs are linked to inflammation and cell apoptosis (C2). (Figure 4c).

The results presented above were validated through Gene Set Enrichment Analysis (GSEA). The gene sets of “Hallmark apoptosis” and “Hallmark inflammatory response” primarily consist of genes that are upregulated by 8-OHD, as evidenced by their high positive normalized enrichment scores (NES) of 2.73 and 2.55, respectively, with a significance level of *p* < 0.001 (Figure 4d). On the other hand, the gene sets associated with “GOBP ribosome biogenesis” (NES= −3.21, *p* < 0.001) and “GOCC ribosome” (NES = −3.01, *p* < 0.001) predominantly contain genes that are downregulated in response to 8-OHD treatment (Figure 4e).

Cancer cells are characterized by intense ribosome biosynthesis; as a result, this feature has become an emerging target for anticancer therapy [20,46,47]. KEGG pathway analysis further showed pathways in ribosome biogenesis (hsa03008, *p* = 4.5 × 10^−11^, 24 genes, Figure 4f) and ribosome (hsa03010, *p* = 2.3 × 10^−7^, 23 genes, Figure 4g) were significantly associated with 8-OHD-downregulated DEGs, as marked in red stars.

### 3.4. AML-Related Genes Are Downregulated by 8-OHD

**Figure 5:**
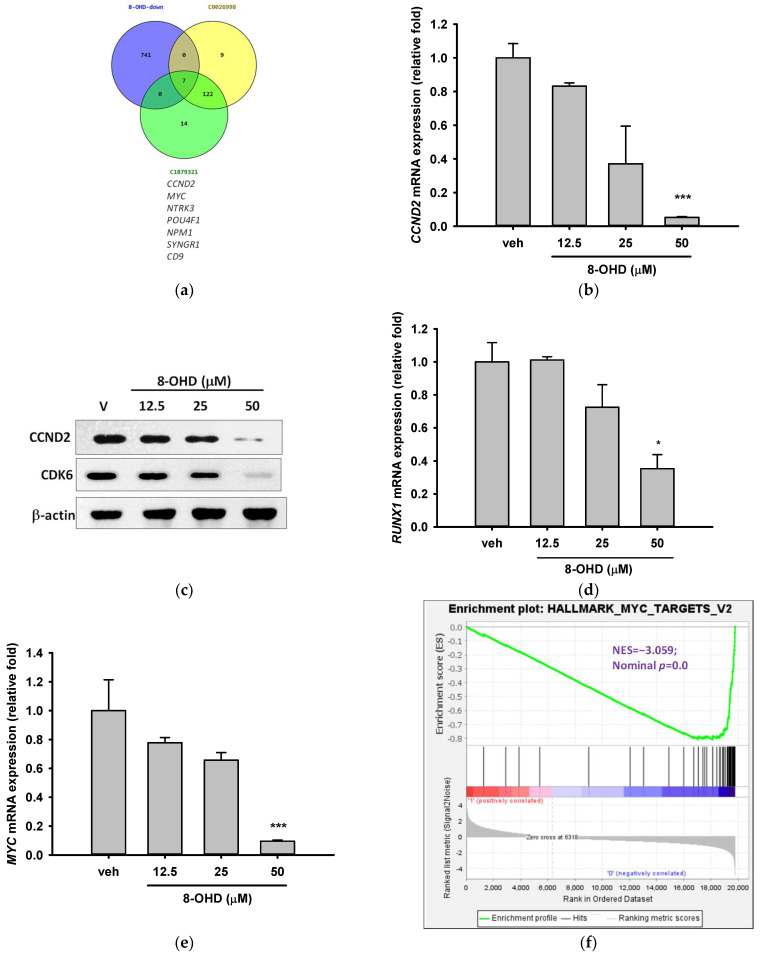

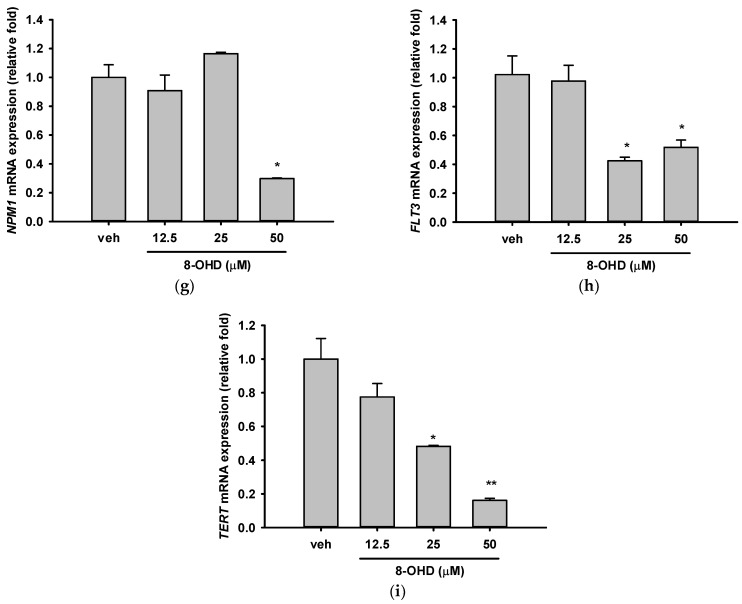
AML-related genes were downregulated by 8-OHD. (**a**) Venn diagram of 8-OHD-downregulated DEGs and gene sets of DisGeNET CUI: C0026998 and CUI: C1879321. The 7 genes in the intersection of three sets are listed below. (**b**,**d**,**e**,**g**–**i**) mRNA expression of AML-targeted genes was measured using RT-qPCR 24 h after treatment, following the methodology outlined in the Materials and Methods section. * *p* < 0.05, ** *p* < 0.01, and *** *p* < 0.001 represent significant differences compared with the vehicle-treated cells. (**c**) Western blotting of CCND2 and CDK6. Total cell lysates were obtained 24 h after 8-OHD treatment, following the protocol outlined in the Materials and Methods section. (**f**) GSEA result indicates that the gene set “Hallmark Myc Targets v. 2” is enriched with 8-OHD-downregulated genes. The barcode plot indicates the position of the genes in each gene set. The horizontal bar in graded color from red to blue indicates up- and down-regulated by 8-OHD. The vertical axis in the lower plot indicates Ranked List Metri.

We then explored the relationship between 8-OHD-downregulated DEGs and AML. By comparing the DEGs with AML gene sets, namely AML-M1, CUI: C0026998 and AML-M2, CUI: C1879321, we identified only seven 8-OHD-downregulated DEGs, including *CCND2*, *MYC*, *NTRK3*, *POU4F1*, *NPM1*, *SYNGR1*, *CD9* were associated with AML (Figure 5a). To obtain more possible target genes, we lowered the stringency by adjusting the threshold of DEG. Supplemental Figure S1 shows the gene intersection between the three gene sets of 8-OHD-downregulated DEGs (V/B > 1.5, *p* < 0.05), C0026998, and C1879321. In addition to the above 7 DEGs, 11 other DEGs were found in the intersection, including *FLT3* and *TERT*. The microarray data in Table 4 reveals the expression profiles of 9 genes, with *CCND2* [48] exhibiting the most downregulation and *TERT* the least.

Cyclin D2, which is encoded by *CCND2*, forms a complex with Cyclin-dependent kinase 4 (CDK4) or CDK6 and functions as a regulatory subunit of the complex, whose activity is required for cell cycle G1/S transition [49,50]. *CCND2* upregulation was found in lots of tumors, including AML [51]. It is well-known that t (8;21) AML is characterized by *CCND2* T280A mutation, which results in a constitutively active cyclin D2 protein and significantly increased cell growth [48]. Table 4 shows that treatment of U-937 cells with 50 μM 8-OHD for 24 h resulted in about 90% decrease in *CCND2* mRNA expression in microarray (*p* = 6.4 × 10^−7^). RT-qPCR reconfirmed that 50 μM 8-OHD significantly reduced *CCND2* mRNA expression by 95% (*p* < 0.001, Figure 5b). Furthermore, Western blot also showed that 8-OHD inhibited CCND2 protein expression dose dependently (Figure 5c).

In addition to its kinase activity, CDK6 regulates cell cycle, apoptosis, stem cell quiescence, differentiation, and inflammation on a transcriptional level [52]. It is often overexpressed in both leukemia and lymphoma [53]. CDK6 was identified as a key regulator of hematopoietic and leukemic stem cell activation [54]; therefore, its inhibition emerges as AML management [52]. Previously, we found that 8-OHD dose dependently inhibited the protein expression of CDK6 and CCND2 and induced cell cycle arrest and apoptosis in K562 CML cells [15]. Figure 5c shows that 8-OHD inhibited CDK6 protein expression in a dose-dependent manner, although it did not significantly inhibit *CDK6* mRNA expression by microarray analysis. Based on the current information, it can be inferred that 8-OHD suppressed the expression of *CCND2*/*CDK6*, which plays a crucial role in regulating the cell cycle and triggered apoptosis in U-937 cells.

It is known that mutant *RUNX1*, a transcription factor, drives *CCND2* expression in AML [55]. Somatic and germline *RUNX1* mutations account for approximately 10% of AML cases [56] and are associated with a poorer outcome [57]. These mutations can enhance self-renewal and block granulocytic differentiation in AML [58]. There are ongoing *RUNX1*-targeted therapies for AML patients with *RUNX1* mutations [59]. Figure 5d shows that treatment of U-937 cells with 50 μM 8-OHD for 24 h resulted in about a 65% decrease in *RUNX1* mRNA expression (*p* < 0.05), although no significant change was noted in microarray analysis. The above information suggests that the induction of U-937 cell apoptosis by 8-OHD may be attributed to its ability to downregulate *RUNX1*/*CCND2*/*CDK6*, which leads to cell cycle arrest.

MYC is a potent driver of ribosome biogenesis and plays a critical role in cell growth and proliferation [60,61]. The literature reports that the upregulation of *MYC* in U-937 cells has the potential to hinder cell differentiation [62]. Table 4 shows that treatment of U-937 cells with 50 μM 8-OHD for 24 h resulted in about 85% decrease in *MYC* mRNA expression in microarray (*p* = 5.0 × 10^−6^). RT-qPCR reconfirmed that 50 μM 8-OHD significantly reduced *MYC* mRNA expression by 90% (*p* < 0.001, Figure 5e). GSEA further supported that gene sets in “Hallmark Myc Targets V2” (NES = −3.06, *p* < 0.001) (Figure 5f) and “Hallmark Myc Targets V1 (NES = −2.60, *p* < 0.001) (Figure S2) were predominantly downregulated by 8-OHD.

Mutations in the *NPM1* and *FLT3* genes are among the most frequently observed genetic alterations in AML patients and have been found to be predictive of therapeutic outcomes and survival [18,63]. They are involved in a variety of functions, such as ribosome biogenesis, chromatin remodeling, and stress responses [64]. *NPM1* gene serves as a transcriptional target of *MYC* [65]. MYC-NPM interaction was found to be essential for the transcription of ribosomal RNA genes and the assembly of ribosomes [66]. Table 4 shows that treatment of U-937 cells with 50 μM 8-OHD for 24 h resulted in about 54% decrease in *NPM1* mRNA expression in microarray (*p* = 1.4 × 10^−4^). RT-qPCR reconfirmed that 50 μM 8-OHD significantly reduced *NPM1* mRNA expression by 70% (*p* < 0.05, Figure 5g). As a result, 50 μM 8-OHD-mediated U-937 cell death may be related to downregulating *MYC-NPM1*-mediated ribosome biogenesis.

FLT3 is a receptor tyrosine kinase that plays a critical role in the development and survival of hematopoietic stem cells, and several FLT3 inhibitors have been developed for AML treatment [67]. Table 4 shows that treatment of U-937 cells with 50 μM 8-OHD for 24 h resulted in about 47% decrease in *FLT3* mRNA expression in microarray (*p* = 0.00025). RT-qPCR reconfirmed that 25 and 50 μM 8-OHD significantly reduced *FLT3* mRNA expression by 50–60% (*p* < 0.05, Figure 5h).

Expression and activation of telomerase reverse transcriptase (TERT) is a key step in AML development [68]. It was reported that *FLT3-ITD* regulates *TERT* expression in AML cells, and treatment of cells with FLT3 inhibitor reduces the expression and activity of TERT in a *MYC*-dependent manner [69]. *TERT* dysregulation by promoter hypermethylation is associated with shorter overall survival and drug resistance in AML patients [70]. The drug 5-azacytidine (5-AZA), which is commonly used for AML treatment, works as a DNA methyltransferase inhibitor and has been shown to lower *TERT* expression and reduce telomerase activity in AML patients and cell lines [71]. Table 4 shows that treatment of U-937 cells with 50 μM 8-OHD for 24 h resulted in about 37% decrease in *TERT* mRNA expression in microarray (*p* = 0.0005). RT-qPCR reconfirmed that 25 and 50 μM 8-OHD significantly reduced *TERT* mRNA expression by 52% (*p* < 0.05) and 84% (*p* < 0.01), respectively (Figure 5i).

We further tested whether 8-OHD also downregulated these genes in THP-1 cells. Supplemental Figure S3 shows that only the expression of *RUNX1*, *MYC*, *NPM-1,* and *TERT* were significantly inhibited by 8-OHD (*p* < 0.05). These suggested that the anti-AML mechanism of 8-OHD is genotype-dependent.

### 3.5. 8-OHD-Upregulated DEGs Are Associated with Inflammatory- and Apoptosis-Related KEGG Pathways

**Figure 6:**
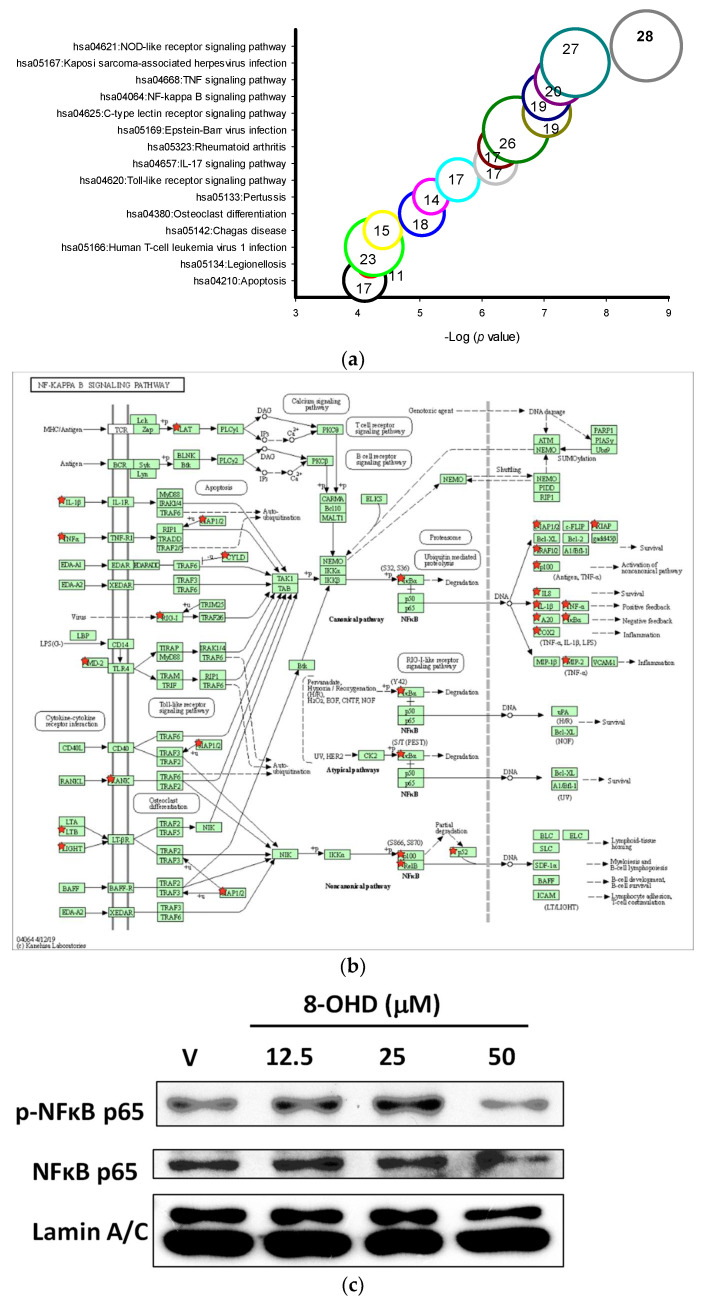

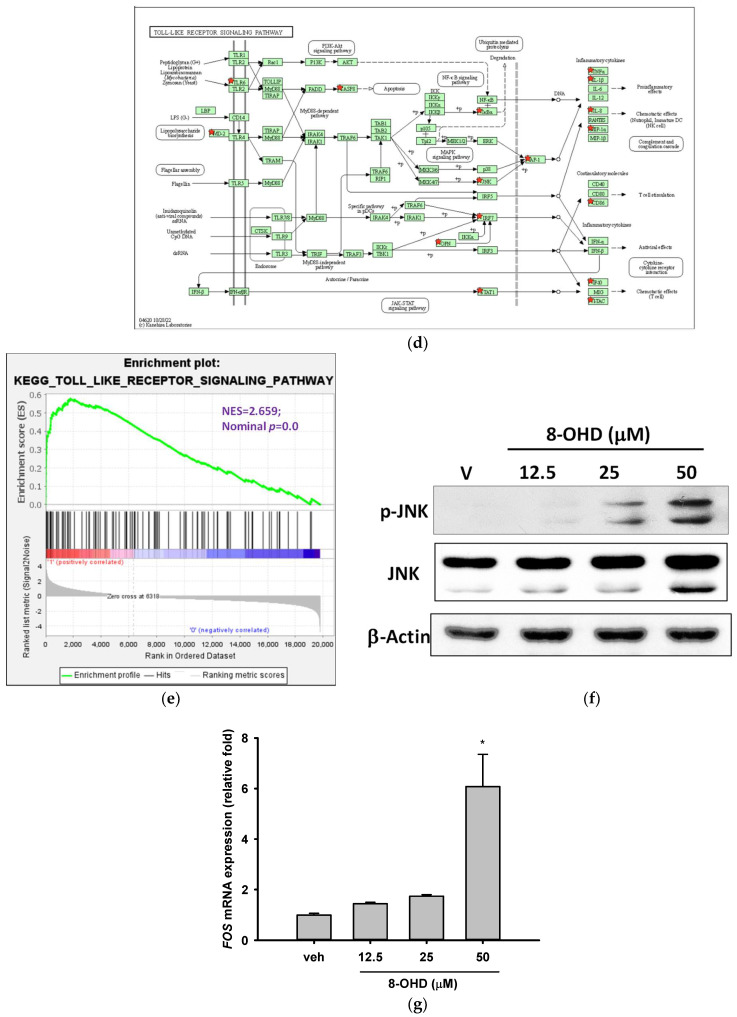

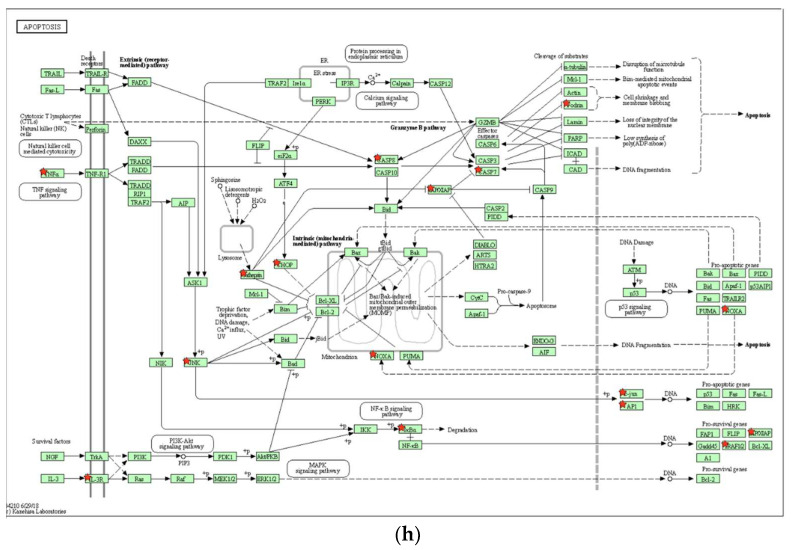
Key KEGG pathways associated with 8-OHD-upregulated DEGs. (**a**) Top 15 enriched KEGG pathways. (**b**) NF-κB signaling pathway (hsa04064). (**c**) Western blot analysis of nuclear NFκB p65 and phospho-NFκB p65. Nuclear extract was prepared 24 h after 8-OHD treatment, as described in Materials and Methods. (**d**) Toll-like receptor signaling pathway (hsa04620). (**e**) GSEA result indicates that the gene set “KEGG Toll-like receptor signaling pathway” is enriched with 8-OHD-upregulated genes. The barcode plot indicates the position of the genes in each gene set. The horizontal bar in graded color from red to blue indicates up- and down-regulated by 8-OHD. The vertical axis in the lower plot indicates Ranked List Metri. (**f**) Western blot analyses of JNK and phospho-JNK. Total cell lysate was prepared 6 h after 8-OHD treatment, as described in Materials and Methods. (**g**) *FOS* gene upregulation. RNA was prepared 24 h after treatment and measured by RT-qPCR as described in Materials and Methods. * *p* < 0.05 represents significant differences compared with the vehicle-treated cells. (**h**) Apoptosis pathway (hsa04210). Upregulated DEGs were marked with red stars in KEGG pathways.

More than 10 KEGG pathways were markedly associated with 8-OHD-upregulated DEGs, such as NOD-like receptor signaling pathway (hsa04621, *p* = 2.2 × 10^−9^, 28 genes), TNF signaling pathway (hsa04668, *p* = 5.4 × 10^−8^, 20 genes), NF-κB signaling pathway (hsa04064, *p* = 8.9 × 10^−8^, 19 genes), Toll-like receptor signaling pathway (hsa04620, *p* = 2.4 × 10^−6^, 17 genes), and Apoptosis (hsa04210, *p* = 7.6 × 10^−5^, 17 genes) (Figure 6a). The five signaling pathways examined here are closely interconnected and share a substantial number of genes, especially those related to inflammation and apoptosis. These findings align with our previous results, which indicated that the “Hallmark apoptosis” and “Hallmark inflammatory response” gene sets are mostly upregulated by 8-OHD, as illustrated in Figure 4d.

NF-κB is a multifunctional transcription factor that mediates pro- and anti-apoptotic as well as pro-autophagic signals [72,73]. Constitutive NF-κB activity has been reported in different types of AML, and it contributes to the resistance to apoptosis [74]. NF-κB signaling-targeted pharmacological approach represented an attractive AML therapeutic strategy [75]. We found that the mRNA expression of the inhibitor of NF-κB, IκBα, was induced by 8-OHD in the NF-κB signaling pathway (hsa04064, up by 2.37-fold, *p* = 0.00013 in microarray, Figure 6b). Western blot showed that nuclear pan-NF-κB and phospho-NF-κB were decreased by 8-OHD (50 μM), indicating that the NF-κB signaling pathway was attenuated in U-937 cells (Figure 6c). However, downstream targets of NF-κB activation, such as TNF-α, COX-2, and IL-1β, were upregulated by 8-OHD by microarray analysis (Figure 6b).

It is well-known that NF-κB and AP-1 are key transcription factors to modulate pro-inflammatory gene expression. From microarray data, it was found that 8-OHD (50 μM) induced expression of *JNK* (up by 2.26-fold, *p* = 6.9 × 10^−5^) and AP1 (*JUN* up by 2.84-fold, *p* = 0.00012; and *FOS* up by 2.28-fold, *p* = 0.00015). In the Toll-like receptor signaling pathway, AP-1 subsequently induced inflammatory cytokine gene upregulation (hsa04620, Figure 6d). GSEA further confirmed that the gene sets in the “Kegg Toll-like receptor signaling pathway” predominantly consisted of 8-OHD-upregulated genes (NES = 2.66, *p* < 0.001) (Figure 6e). We then performed Western blot and RT-qPCR to confirm that 8-OHD treatment significantly increased JNK expression and phosphorylation as well as *FOS* expression as compared with vehicle, respectively (Figure 6f,g). It is possible that 8-OHD upregulates inflammatory gene expression through the JNK-dependent AP-1 pathway in U-937 cells.

TNF-α is a central mediator of cytokine cascade and is a highly pleiotropic cytokine working through binding to two distinct receptors: TNF-R1 and TNF-R2. Generally speaking, TNF-α relies on TNFR1 for apoptosis and TNFR2 for survival [76]. The apoptosis pathway (hsa04210) clearly shows that binding of TNF-α to TNF-R1 induces receptor trimerization and recruitment of TNFR1-associated death domain protein (TRADD) and subsequent Fas-associated death domain protein (FADD). This complex transmits an activating signal from the activated receptor TNFR1 to a caspase cascade with subsequent apoptosis (Figure 6h). From microarray data, we found that 8-OHD upregulates the *TNF* gene by 5.39-fold (*p* = 9.8 × 10^−4^), the initiator *CASP8* by 2.27-fold (*p* = 0.00038), and the subsequent effector *CASP7* by 2.04-fold (*p* = 0.00047).

In conclusion, current data suggests that 8-OHD can stimulate JNK expression/phosphorylation and AP-1 overexpression, which in turn causes overexpression of TNF-α. This overexpression triggers overexpression and activation of caspase-7 and cleavage of PARP-1 (as shown in Figure 3), ultimately leading to apoptosis in U-937 cells.

### 3.6. In Silico Prediction of Potential Protein Targets of 8-OHD

**Figure 7:**
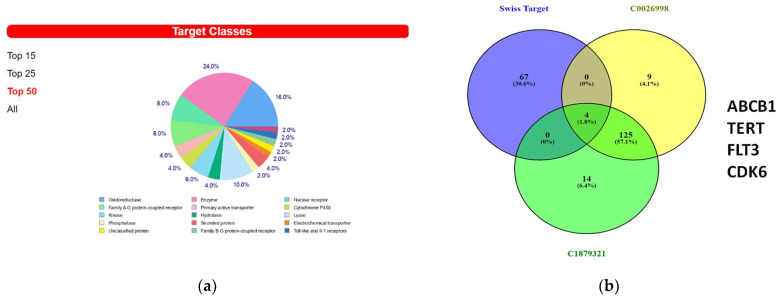
Predicted 8-OHD binding targets using Swiss Target Prediction. (**a**) The summary of the predicted target classes displayed as a pie chart. Percentages are calculated using the top 50 predicted targets. (**b**) Venn diagram of predicted 8-OHD targets and gene sets of DisGeNET CUI: C0026998 and CUI: C1879321. The 4 targets in the intersection of three sets were listed.

We then used the Swiss Target Prediction website to find out the possible protein targets of 8-OHD. Supplemental Table S3 shows the list of 71 predicted target proteins with a probability greater than 0. The Top 50 targets consisted mostly of enzymes such as oxidoreductases, kinases, hydrolases, lysases, and phosphatases. Receptors, including nuclear receptors, G-protein coupled receptors, and Toll-like receptors, were also among the identified targets (Figure 7a).

A Venn diagram analysis of predicted 8-OHD protein targets, C0026998 and C1879321, was performed. Only four proteins, ABCB1, TERT, FLT3, and CDK6, were found in the overlapping part (Figure 7b). The lists of known actives similar in 3D to 8-OHD on TERT, FLT3, and CDK6 were shown in Supplemental Figure S4. It appears that only flavones, rather than isoflavones, could be found.

**Figure 8:**
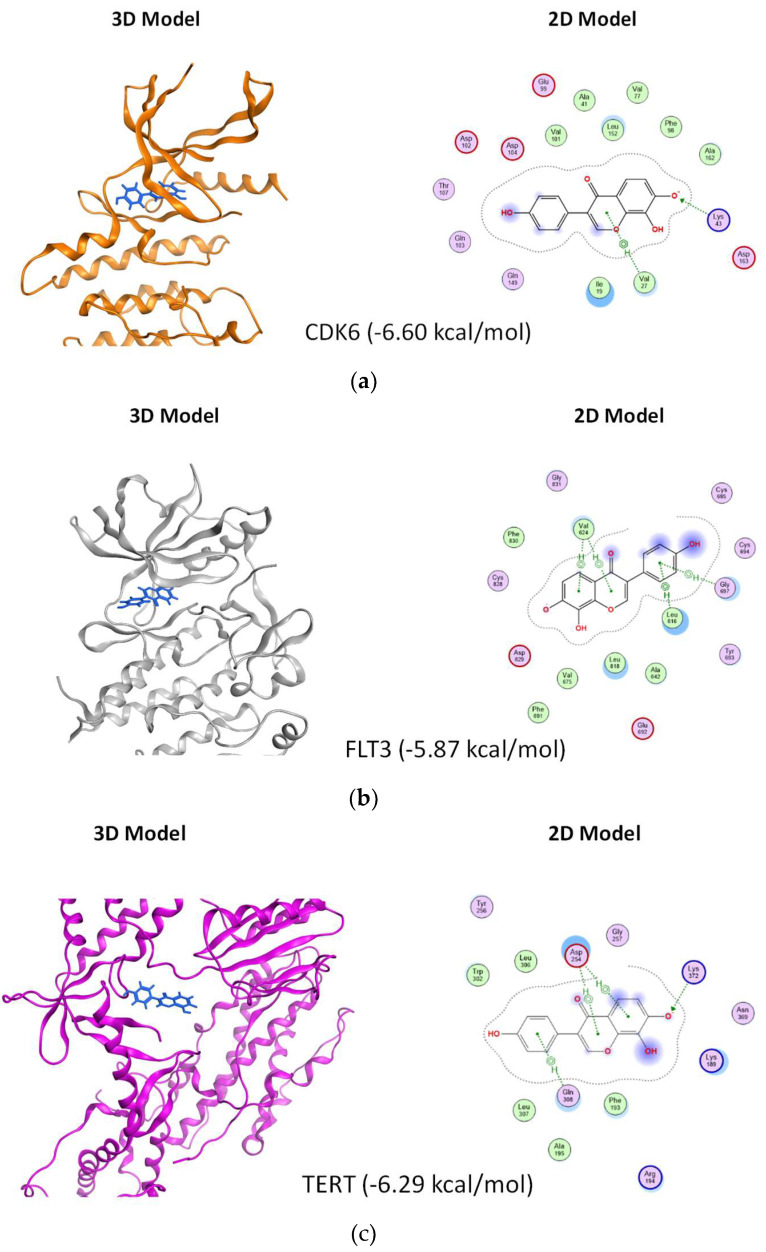
Plausible docking models of 8-OHD with AML target proteins. The preferable 3D and 2D poses of 8-OHD docked into the binding domain of different proteins. (**a**) CDK6 (docking score: −6.60). (**b**) FLT3 (docking score: −5.87). (**c**) TERT (docking score: −6.29). The proteins are shown in ribbon, and the compound is shown in blue color in 3D model. In 2D model, key amino acids within 4.5 Å of docked 8-OHD and their binding interactions were identified. Polar residues are colored mauve, while hydrophobic residues are represented in green. Basic residues are indicated with a blue rim and acidic residues with a red rim. Hydrogen bonds are represented as dotted lines with arrows indicating the direction of the bond. A halo-like disc around a residue denotes a reduction in solvent exposure induced by 8-OHD [77]. CH/π hydrogen bond interactions are shown by a dotted green line from the residue to the center of the ring of 8-OHD.

To enhance our understanding of the interaction between isoflavone 8-OHD and its three targets, we utilized the Molecular Operating Environment (MOE) software and applied the GBVI/WSA dG scoring function to assess the free energy involved in the binding process of 8-OHD to each target. According to the findings, the docking scores for the interactions of 8-OHD with CDK6, FLT3, and TERT were −6.60 kcal/mol, −5.87 kcal/mol, and −6.29 kcal/mol, respectively. The results indicate that 8-OHD might have a higher binding affinity towards CDK6 than the other two proteins (Figure 8a–c). Upon analyzing the ligand–protein interaction map of 8-OHD with CDK6, it was observed that the phenol group of 8-OHD displayed a preference towards binding with hydrophilic residues (mauve interior) within the binding pocket, whereas other regions of the compound tended to interact with the hydrophobic amino acids (green interior) (Figure 8a). However, in the case of 8-OHD binding to FLT3 and TERT proteins, the phenol group displayed a tendency to interact with less hydrophilic residues, which might explain why 8-OHD exhibited a higher binding preference towards CDK6 as compared to the other two proteins (Figure 8b,c). In conclusion, the findings suggest that 8-OHD is capable of interacting with CDK6, TERT, and FLT3 proteins with differing affinities, thereby leading to varying degrees of reduction in their enzyme activities. The mechanism described above, in addition to the gene downregulation mentioned previously, leads to the inhibition of U-937 cell proliferation.

### 3.7. Synergic Pharmacological Interaction between 8-OHD and Cytarabine (Ara-C)

**Figure 9:**
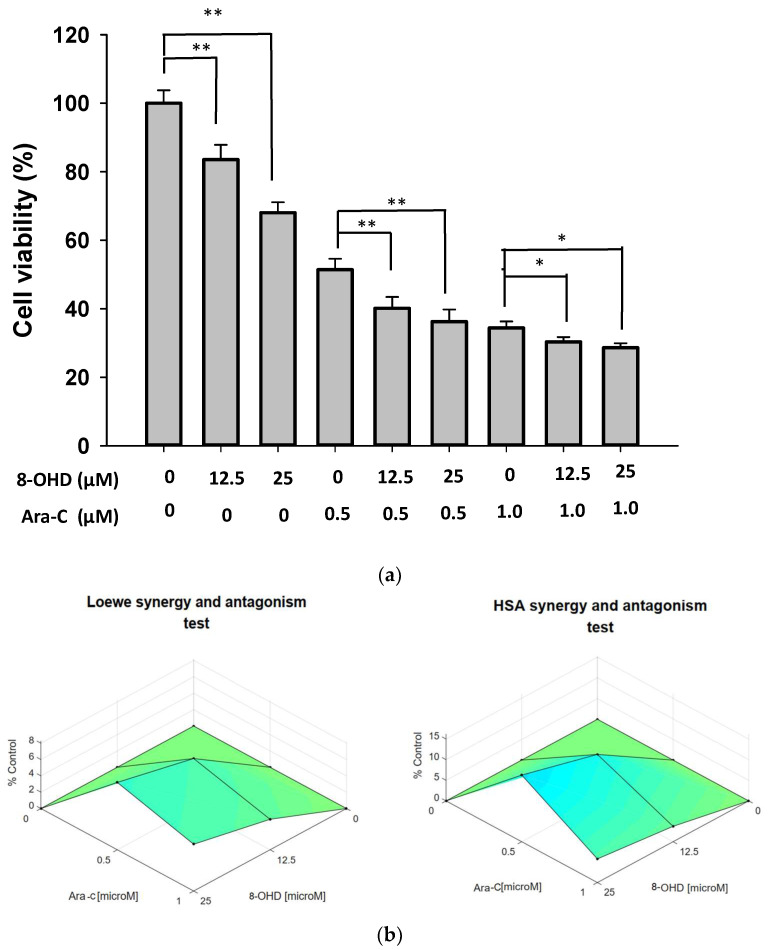
Synergic effect of combination treatment of 8-OHD and Ara-C in U-937 cells. (**a**) U-937 cells were treated with vehicle (0.1% DMSO), 8-OHD, Ara-C, or combination for 24 h. Cell viability was examined using trypan blue exclusion test. The experiments were repeated three times. These data represent the mean ± SD of three independent experiments. * *p* < 0.05 and ** *p* < 0.01 represent significant differences compared with indicated treatment. (**b**) Synergy scores displayed in Surface plots modeled by Loewe and HSA.

Induction therapy with cytarabine (Ara-C) and anthracycline remains a standard treatment in AML. We further examined the combination effect of 8-OHD and Ara-C in U-937 cells. Figure 9a shows that Ara-C (0.5 or 1 μM) alone exhibited strong and dose-dependent cytotoxicity as detected by trypan blue exclusion analysis. Co-treatment of U-937 with Ara-C (0.5 μM) and 8-OHD (12.5 or 25 μM) resulted in a significantly higher reduction in cell viability as compared with Ara-C- or 8-OHD-treated cells (*p* < 0.01). Less significance was noted for co-treatment with higher concentrations of Ara-C (1.0 μM) and 8-OHD (*p* < 0.05). Synergy scores were computed using Combenefit_2_02 free software [37]. A synergistic pharmacological interaction was evident between 8-OHD and Ara-C as the synergy scores were displayed as Surface plots modeled by the Loewe and Highest Single Agent (HAS) (*p* < 0.05) (Figure 9b).

The cytotoxic action of the prodrug Ara-C relies on its enzymatic conversion into the active triphosphorylated form, Ara-CTP. Several genes, including human equilibrative nucleoside transporter-1 (*ENT1*), deoxycytidine kinase (*DCK*), cytidine deaminase (*CDA*), and the subunits of ribonucleotide reductase (RRM1 and RRM2), play crucial roles in uptake and metabolizing Ara-C as well as keeping dNTP homeostasis, and are valuable predictors of ex vivo drug response as well as treatment outcomes [78,79]. Elevated activity and expression levels of *CDA* have been observed in AML patients who do not respond to induction chemotherapy [80]. Conversely, reduced expression and activity of *DCK* in AML have been identified as a mechanism contributing to clinical resistance against Ara-C [81]. AML samples with increased mRNA expression of *DCK*, *ENT1*, and *RRM1*, coupled with decreased *CDA* expression, have been found to exhibit sensitivity to Ara-C in ex vivo cytotoxicity assays [78]. From microarray data, we found that *CDA* was significantly downregulated by 8-OHD, while *DCK* and *RRM2* were markedly upregulated by 8-OHD in U-937 cells (Supplemental Table S4). These gene expression changes may be responsible for sensitizing U-937 cells to Ara-C treatment.

## 4. Conclusions

Our study presents in vitro evidence of the anti-AML properties of 8-OHD and sheds light on the underlying molecular mechanisms involved. Through our experiments, we observed a significant and dose- and time-dependent inhibition of U-937 cell viability by 8-OHD. This inhibition was accompanied by the downregulation of cyclin D (CCND2) and cyclin D-dependent kinase (CDK6) and the activation of JNK and AP-1 overexpression, ultimately leading to caspase-7-dependent apoptosis.

To gain a deeper understanding of the effects of 8-OHD in U-937 cells, we conducted a comprehensive microarray-based transcriptome profiling. This analysis revealed 676 upregulated differentially expressed genes (DEGs) and 748 downregulated DEGs in U-937 cells after 24 h of incubation with 8-OHD (50 μM). Functional enrichment analysis demonstrated that the downregulated DEGs were significantly associated with gene ontology (GO) terms related to rRNA processing and ribosome biogenesis. Conversely, the upregulated DEGs were linked to inflammation and cell apoptosis, which was further validated using Gene Set Enrichment Analysis (GSEA).

Furthermore, our investigation revealed the downregulation of AML-target genes, namely *CCND2*, *MYC*, *NPM1*, *FLT3*, and *TERT*, upon treatment with 8-OHD, as confirmed by RT-qPCR. GSEA analysis substantiated that the gene set “Hallmark Myc Targets” was predominantly downregulated by 8-OHD, highlighting the central role of Myc in this context. Additionally, molecular docking studies indicated that 8-OHD interacts with CDK6, TERT, and FLT3, and these interactions likely lead to the inhibition of cell signaling, cell cycle progression, and proliferation.

Importantly, we observed a synergic cytotoxic effect when 8-OHD was combined with Ara-C in U-937 cells. This finding suggests the potential of utilizing 8-OHD in combination therapy with Ara-C, which could enhance its therapeutic efficacy. Overall, our study provides crucial insights into the anti-AML activities of 8-OHD and the underlying molecular mechanisms. 8-OHD shows promise as a maintenance therapy option for preventing disease relapse by effectively modulating the expression of multiple AML-related genes, including those associated with Ara-C resistance.

## Figures and Tables

**Table 1:** Primary antibodies used in Western blotting.

Antibody	Company	Catalog Number
β-Actin	GeneTex (Irvine, CA, USA)	629630
α-Tubulin	Sigma–Aldrich	T6199
PARP-1	Santa Cruz Biotechnology (Santa Cruz, CA, USA)	7150
Lamin A/C	Genetex	101127
Phospho-JNK1/2	Cell Signaling (Danvers, MA, USA)	4668
JNK2	Cell Signaling	9258
Phospho-p65 NF-κB	Cell Signaling	3033
p65 NF-κB	Cell Signaling	8242
Caspase-7	Cell Signaling	9492
CDK6	Cell Signaling	13331
CCND2	Cell Signaling	3742

**Table 2:** The primer pairs used in qPCR.

Gene	Primer Sequence (5′-3′)		Size (bp)
*GAPDH*	F	CATGAGAAGTATGACAACAGCCT	113
	R	AGTCCTTCCACGATACCAAAGT	
*CCND2*	F	TTTGCCATGTACCCACCGTC	104
	R	AGGGCATCACAAGTGAGCG	
*FLT3*	F	CGGGCTCACCTGGGAATTAG	130
	R	GTCGTTTCTTGCCACTGATGA	
*MYC*	F	GTCAAGAGGCGAACACACAAC	162
	R	TTGGACGGACAGGATGTATGC	
*NPM1*	F	GGAGGTGGTAGCAAGGTTCC	143
	R	TTCACTGGCGCTTTTTCTTCA	
*RUNX1*	F	CTTGTCTCCACTGAGGCACA	133
	R	CTGTGTAGGGGAGCCACATT	
*TERT*	F	CCGATTGTGAACATGGACTACG	99
	R	CACGCTGAACAGTGCCTTC	
*FOS*	F	GCCTCTCTTACTACCACTCACC	126
	R	AGATGGCAGTGACCGTGGGAAT	

**Table 3:** Estimated IC_50_ values of 8-OHD in three cell lines at 24 and 48 h.

Cell Line	IC_50_ (μM) ± SD		
	U-937	THP-1	HL-60
24 h	84.5 ± 3.8	17.3 ± 0.9	32.5 ± 1.6
48 h	32.5 ± 1.0	11.3 ± 0.5	24.4 ± 0.6

**Table 4:** Selected 8-OHD-downregulated genes associated with DisGeNET AML.

Entrez Gene	Gene Symbol	log_2_FC	*p* Value
894	*CCND2*	−3.28	6.38 × 10^−7^
4609	*MYC*	−2.81	4.99 × 10^−6^
4916	*NTRK3*	−1.96	2.06 × 10^−5^
5457	*POU4F1*	−1.79	9.97 × 10^−6^
4869	*NPM1*	−1.12	1.40 × 10^−4^
9145	*SYNGR1*	−1.11	1.94 × 10^−4^
928	*CD9*	−1.04	2.46 × 10^−4^
2322	*FLT3*	−0.92	2.48 × 10^−4^
7015	*TERT*	−0.66	4.98 × 10^−3^

## Data Availability

The data presented in this study are available in this article (and Supplementary Material).

## Supplementary Materials

The following supporting information can be downloaded at: https://www.mdpi.com/article/10.3390/biom13111575/s1, Table S1. Enriched GO terms with 8-OHD-downregulated DEGs (adjusted *p* < 0.05); Table S2. Enriched GO terms with 8-OHD-upregulated DEGs (adjusted *p* < 0.05); Table S3. 8-OHD Target Protein Prediction; Table S4. 8-OHD affects Ara-C-metabolizing gene expression; Figure S1. Venn diagram of 8-OHD-downregulated DEGs (B/V > 1.5 and *p* < 0.05) and gene sets of DisGeNET CUI: C0026998 and CUI: C1879321; Figure S2. Effects of 8-OHD on the expression of *RUNX1*, *MYC*, *NPM1,* and *TERT* in THP-1 cells; Figure S3. GSEA result indicates that the gene set “Hallmark Myc Targets v.1” is enriched with 8-OHD-downregulated DEGs; Figure S4. Known actives on TERT, FLT3, and CDK6 that are highly similar to 8-OHD according to 3D measures.
