# Supplementary material for: 8-Hydroxydaidzein Induces Apoptosis and Inhibits AML-Associated Gene Expression in U-937 Cells: Potential Phytochemical for AML Treatment
Source: Biomolecules. 2023 Oct 26;13(11):1575. doi: 10.3390/biom13111575 (PMC10669020; doi:10.3390/biom13111575)
Supplement: Supplementary file 1 [file biomolecules-13-01575-s001.zip › biomolecules-2630681-original-images.pdf]

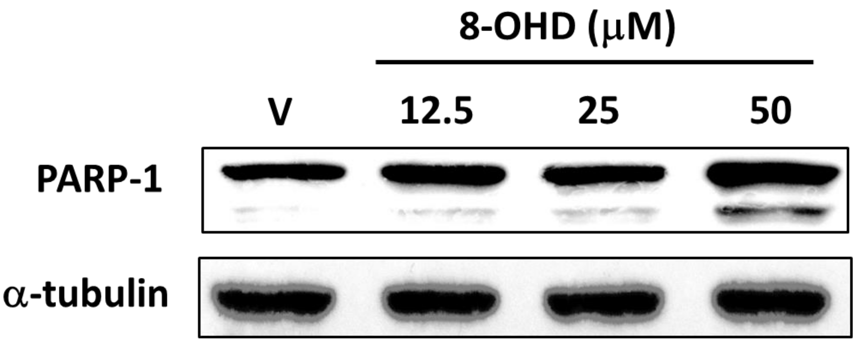

Fig3a.

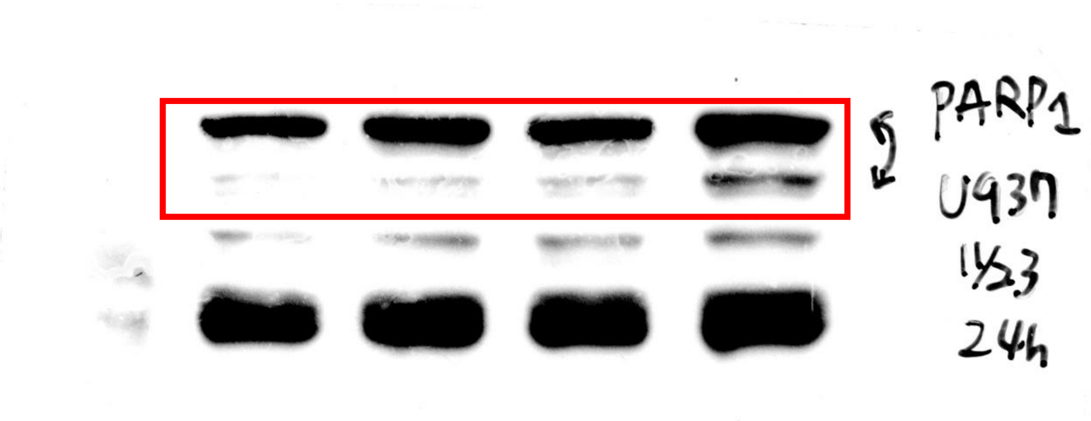

PARA-1

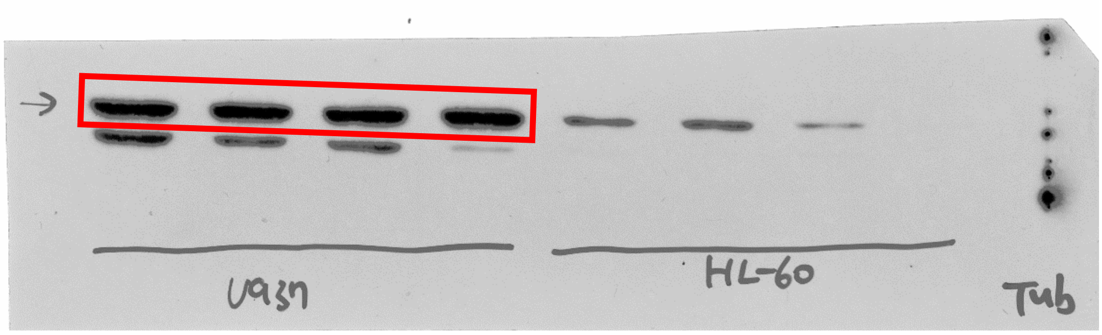

Tubulin

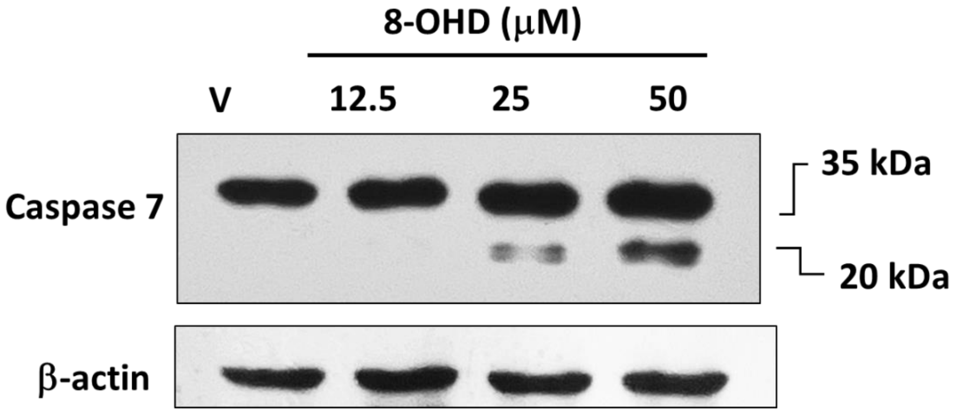

Fig 3b

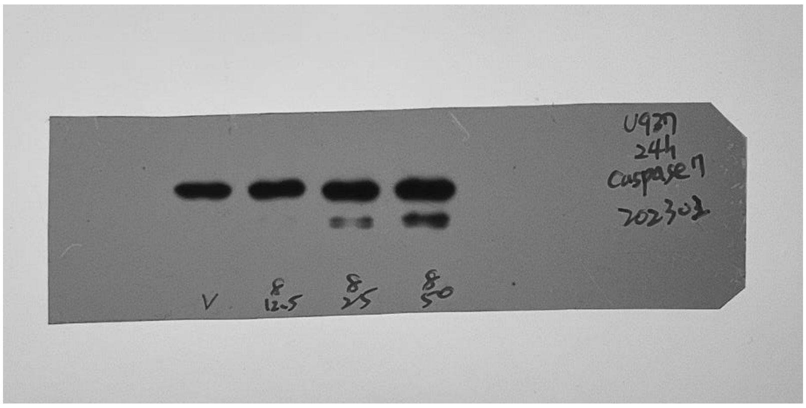

Caspase 7

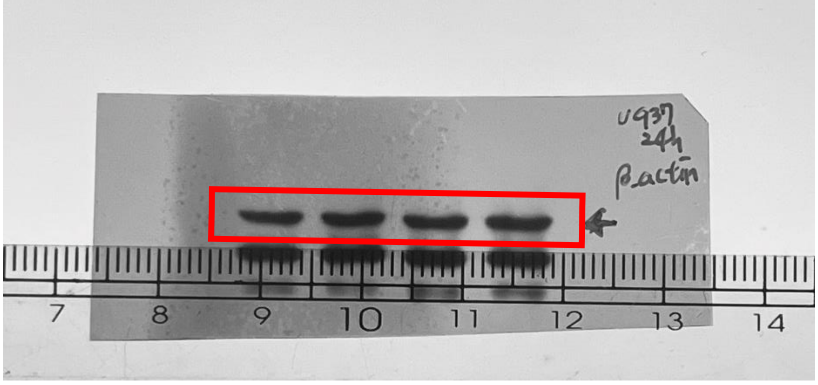

β-actin

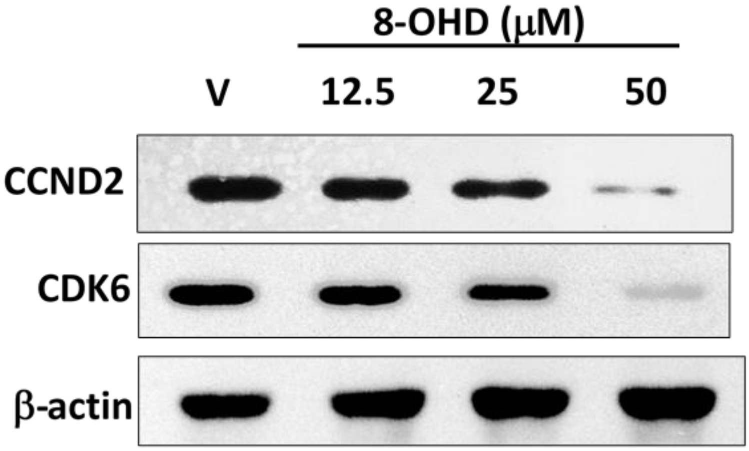

(c)

Fig. 5c

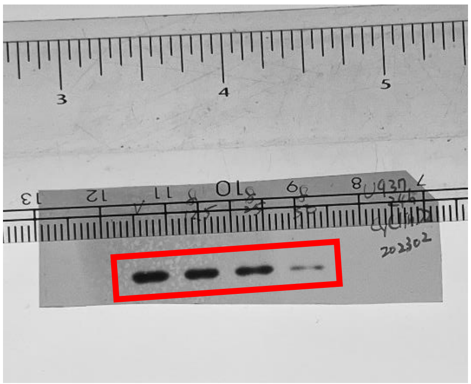

CCND2

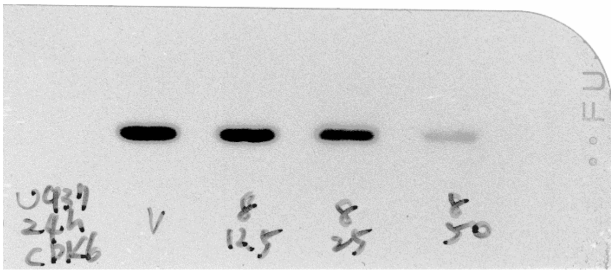

CDK6

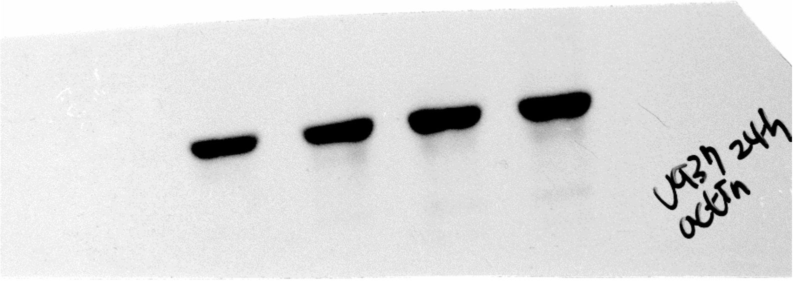

β-actin

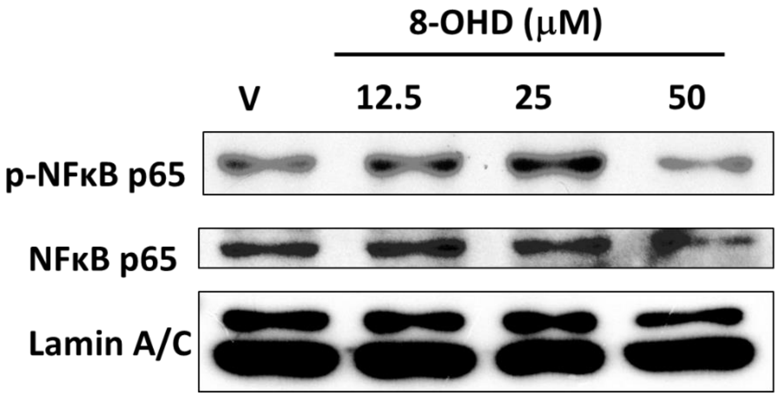

Fig 6c

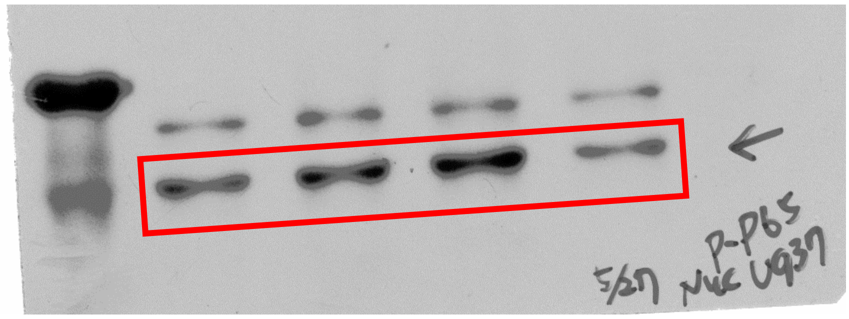

p-NFkB p65

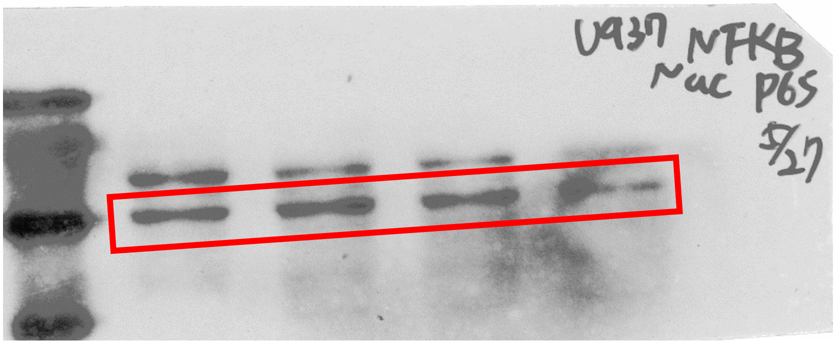

NFkB p65

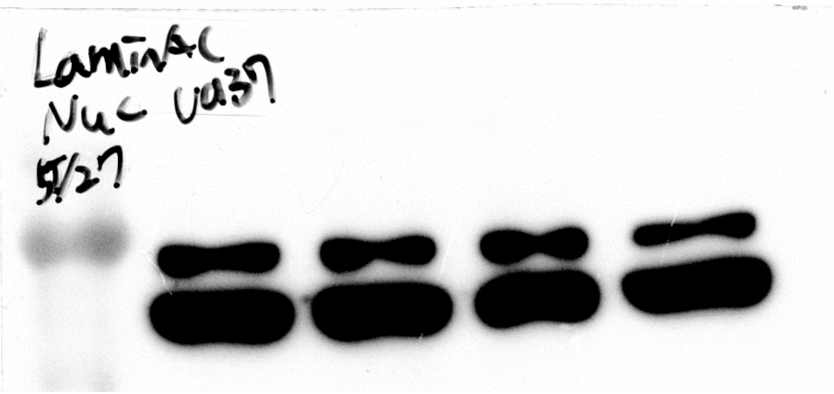

Lamin A/C

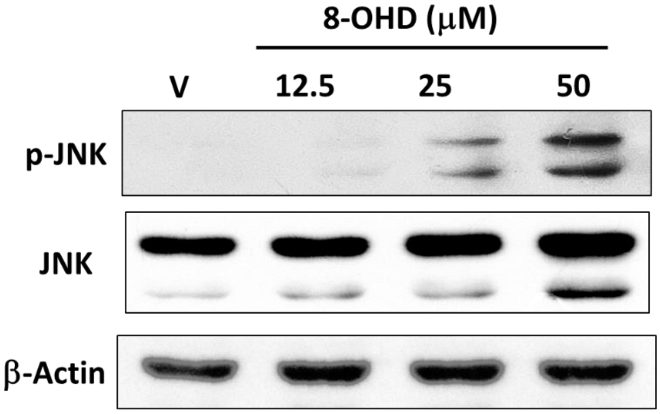

Fig 6f

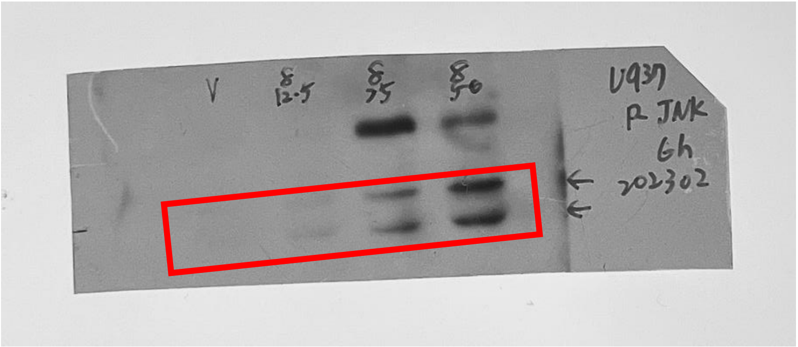

p-JNK

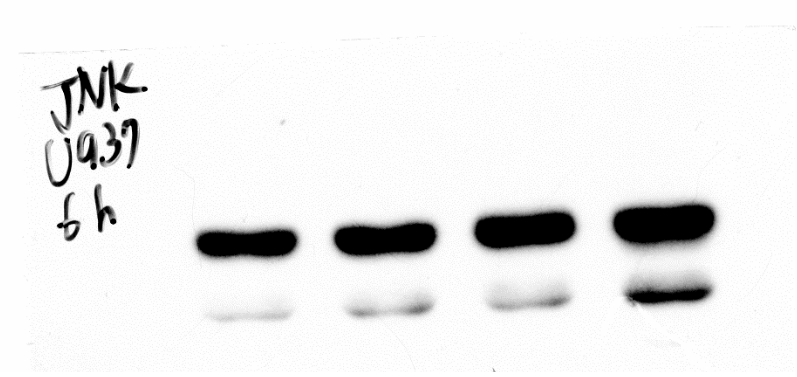

JNK

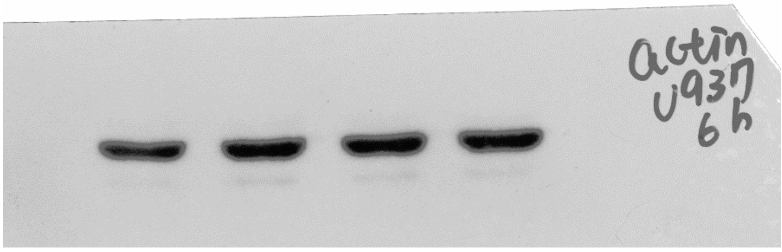

β-actin
