# Supplementary material for: 8-Hydroxydaidzein Induces Apoptosis and Inhibits AML-Associated Gene Expression in U-937 Cells: Potential Phytochemical for AML Treatment
Source: Biomolecules. 2023 Oct 26;13(11):1575. doi: 10.3390/biom13111575 (PMC10669020; doi:10.3390/biom13111575)
Supplement: Supplementary file 1 [file biomolecules-13-01575-s001.zip › biomolecules-2630681-supplementary.pdf]

Supplemental Materials

Table S1. Enriched GO terms with 8-OHD-downregulated DEGs (adjusted p<0.05).

c1	Ontology Type	Ontology Name(Ontology-ID)	Number Changed	Number Measured	Number in Ontology	Percent Changed	Z Score	Fisher Exact P	Adjusted P
c1	BP	rRNA processing(GO:0006364)	32	120	120	26.7	15.27	8.82E-22	3.46E-18
c1	CC	nucleolus(GO:0005730)	75	697	697	10.8	12.25	3.45E-22	1.81E-18
c1	BP	ribosome biogenesis(GO:0042254)	15	52	52	28.8	10.96	1.85E-11	3.24E-08
c1	BP	pseudouridine synthesis(GO:0001522)	9	20	20	45.0	11.04	2.25E-09	1.86E-06
c1	CC	mitochondrion(GO:0005739)	86	1512	1512	5.7	6.40	6.63E-09	4.96E-06
c1	MF	pseudouridine synthase activity(GO:0009982)	7	16	16	43.8	9.58	1.87E-07	9.78E-05
c1	CC	small nucleolar ribonucleoprotein complex(GO:0005732)	6	14	14	42.9	8.76	1.70E-06	7.05E-04
c1	CC	DNA-directed RNA polymerase I complex(GO:0005736)	4	5	5	80.0	10.11	3.86E-06	1.48E-03
c1	CC	mitochondrial part(GO:0044429)	49	835	835	5.9	4.99	9.45E-06	3.37E-03
c1	BP	tRNA modification(GO:0006400)	7	28	28	25.0	6.84	1.41E-05	4.88E-03
c1	BP	translational elongation(GO:0006414)	14	120	120	11.7	5.60	1.43E-05	4.88E-03
c1	BP	translation(GO:0006412)	24	310	310	7.7	4.95	2.26E-05	7.40E-03
c1	CC	ribosome(GO:0005840)	20	233	233	8.6	5.04	2.51E-05	8.05E-03
c1	CC	preribosome, small subunit precursor(GO:0030688)	3	3	3	100.0	9.87	2.66E-05	8.20E-03
c1	MF	transferase activity, transferring one-carbon groups(GO:0016741)	19	215	215	8.8	5.06	2.66E-05	8.20E-03
c1	BP	sulfur compound biosynthetic process(GO:0044272)	13	114	114	11.4	5.29	3.66E-05	1.09E-02
c1	MF	DNA-directed RNA polymerase activity(GO:0003899)	9	56	56	16.1	5.76	3.89E-05	1.11E-02
c1	BP	viral transcription(GO:0019083)	12	103	103	11.7	5.17	5.87E-05	1.59E-02
c1	BP	gene expression(GO:0010467)	44	778	778	5.7	4.45	5.67E-05	1.59E-02
c1	MF	structural constituent of ribosome(GO:0003735)	17	193	193	8.8	4.77	7.26E-05	1.87E-02
c1	MF	snoRNA binding(GO:0030515)	5	16	16	31.3	6.64	7.80E-05	1.95E-02
c1	CC	PeBoW complex(GO:0070545)	3	4	4	75.0	8.46	1.04E-04	2.35E-02
c1	BP	RNA catabolic process(GO:0006401)	20	258	258	7.8	4.52	1.05E-04	2.35E-02

Table S2. Enriched GO terms with 8-OHD-upregulated DEGs (adjusted *p*<0.05).

c2	Ontology Type	Ontology Name(Ontology-ID)	Number Changed	Number Measured	Number in Ontology	Percent Changed	Z Score	Fisher Exact P	Adjusted P
c2	BP	response to biotic stimulus(GO:0009607)	75	821	821	9.1	11.91	2.32E-21	3.65E-17
c2	BP	immune response(GO:0006955)	81	1216	1216	6.7	9.08	1.12E-14	2.20E-11
c2	BP	inflammatory response(GO:0006954)	41	418	418	9.8	9.28	4.29E-13	5.19E-10
c2	BP	positive regulation of defense response(GO:0031349)	31	248	248	12.5	9.79	6.15E-13	6.90E-10
c2	BP	leukocyte activation(GO:0045321)	36	369	369	9.8	8.65	1.41E-11	1.11E-08
c2	BP	regulation of apoptosis(GO:0042981)	75	1342	1342	5.6	7.02	4.87E-10	2.32E-07
c2	BP	leukocyte migration(GO:0050900)	24	227	227	10.6	7.53	7.79E-09	2.48E-06
c2	BP	positive regulation of programmed cell death(GO:0043068)	41	575	575	7.1	6.86	7.90E-09	2.48E-06
c2	BP	negative regulation of molecular function(GO:0044092)	49	789	789	6.2	6.42	3.54E-08	9.11E-06
c2	BP	regulation of sequence-specific DNA binding transcription factor activity(GO:0051090)	29	365	365	7.9	6.42	1.36E-07	3.01E-05
c2	BP	cellular response to stress(GO:0033554)	62	1174	1174	5.3	5.86	1.49E-07	3.25E-05
c2	CC	cytosol(GO:0005829)	113	2666	2666	4.2	5.58	1.71E-07	3.63E-05
c2	BP	regulation of protein kinase activity(GO:0045859)	43	708	708	6.1	5.84	4.24E-07	8.21E-05
c2	BP	response to mechanical stimulus(GO:0009612)	17	150	150	11.3	6.70	4.45E-07	8.43E-05
c2	BP	negative regulation of response to stimulus(GO:0048585)	45	770	770	5.8	5.70	7.21E-07	1.33E-04
c2	BP	regulation of signal transduction(GO:0009966)	81	1791	1791	4.5	5.26	1.29E-06	2.30E-04
c2	BP	negative regulation of viral genome replication(GO:0045071)	9	43	43	20.9	7.52	1.41E-06	2.42E-04
c2	BP	cell cycle arrest(GO:0007050)	15	143	143	10.5	5.90	5.55E-06	7.39E-04
c2	BP	positive regulation of cytokine production(GO:0001819)	20	240	240	8.3	5.57	5.89E-06	7.71E-04
c2	BP	regulation of biological quality(GO:0065008)	99	2417	2417	4.1	4.81	6.87E-06	8.85E-04
c2	BP	response to organic cyclic compound(GO:0014070)	19	224	224	8.5	5.51	7.78E-06	9.86E-04
c2	BP	negative regulation of immune system process(GO:0002683)	19	225	225	8.4	5.49	8.29E-06	1.04E-03
c2	BP	positive regulation of metabolic process(GO:0009893)	85	2005	2005	4.2	4.76	8.95E-06	1.12E-03

c2	BP	regulation of transcription from RNA polymerase II promoter(GO:0006357)	64	1393	1393	4.6	4.76	1.18E-05	1.45E-03
c2	BP	developmental maturation(GO:0021700)	14	137	137	10.2	5.58	1.53E-05	1.85E-03
c2	MF	chemokine activity(GO:0008009)	9	57	57	15.8	6.23	1.62E-05	1.91E-03
c2	MF	cyclin-dependent protein kinase regulator activity(GO:0016538)	6	22	22	27.3	7.24	1.66E-05	1.94E-03
c2	MF	2'-5'-oligoadenylate synthetase activity(GO:0001730)	3	3	3	100.0	10.55	1.80E-05	2.09E-03
c2	BP	response to metal ion(GO:0010038)	19	239	239	7.9	5.18	1.95E-05	2.22E-03
c2	MF	DNA binding(GO:0003677)	103	2633	2633	3.9	4.42	2.74E-05	2.99E-03
c2	BP	transforming growth factor beta receptor signaling pathway(GO:0007179)	13	126	126	10.3	5.42	2.77E-05	3.00E-03
c2	BP	cellular response to external stimulus(GO:0071496)	17	207	207	8.2	5.05	3.51E-05	3.58E-03
c2	CC	nucleus(GO:0005634)	193	5712	5712	3.4	4.19	4.30E-05	4.31E-03
c2	BP	negative regulation of signaling(GO:0023057)	35	640	640	5.5	4.57	4.38E-05	4.33E-03
c2	BP	positive regulation of prostaglandin secretion(GO:0032308)	4	9	9	44.4	7.85	5.32E-05	5.10E-03
c2	BP	regulation of heat generation(GO:0031650)	5	17	17	29.4	6.91	5.82E-05	5.38E-03
c2	BP	negative regulation of plasminogen activation(GO:0010757)	3	4	4	75.0	9.06	7.05E-05	6.44E-03
c2	BP	plasma membrane organization(GO:0007009)	9	69	69	13.0	5.42	7.72E-05	6.94E-03
c2	BP	response to temperature stimulus(GO:0009266)	12	123	123	9.8	4.96	9.67E-05	8.26E-03
c2	BP	regulation of adaptive immune response(GO:0002819)	12	125	125	9.6	4.89	1.13E-04	9.35E-03
c2	BP	regulation of angiogenesis(GO:0045765)	14	166	166	8.4	4.70	1.28E-04	1.03E-02
c2	BP	negative regulation of cell communication(GO:0010648)	34	643	643	5.3	4.29	1.27E-04	1.03E-02
c2	BP	lymphocyte mediated immunity(GO:0002449)	9	74	74	12.2	5.14	1.34E-04	1.07E-02
c2	CC	cytoplasm(GO:0005737)	172	5082	5082	3.4	3.90	1.48E-04	1.17E-02
c2	BP	regulation of NF-kappaB import into nucleus(GO:0042345)	7	45	45	15.6	5.43	1.58E-04	1.22E-02
c2	CC	I-kappaB/NF-kappaB complex(GO:0033256)	3	5	5	60.0	8.03	1.73E-04	1.31E-02
c2	BP	regulation of defense response to virus by host(GO:0050691)	6	33	33	18.2	5.60	1.92E-04	1.43E-02

c2	BP	regulation of interleukin-6 production(GO:0032675)	9	78	78	11.5	4.93	2.02E-04	1.47E-02
c2	BP	regulation of type I interferon production(GO:0032479)	9	78	78	11.5	4.93	2.02E-04	1.47E-02
c2	MF	double-stranded RNA binding(GO:0003725)	7	47	47	14.9	5.27	2.09E-04	1.52E-02
c2	BP	negative regulation of developmental process(GO:0051093)	32	607	607	5.3	4.14	2.33E-04	1.64E-02
c2	BP	hemopoietic or lymphoid organ development(GO:0048534)	15	198	198	7.6	4.38	2.43E-04	1.69E-02
c2	BP	integrin-mediated signaling pathway(GO:0007229)	9	80	80	11.3	4.84	2.45E-04	1.69E-02
c2	BP	response to interferon-beta(GO:0035456)	4	13	13	30.8	6.35	2.78E-04	1.87E-02
c2	BP	regulation of vascular endothelial growth factor production(GO:0010574)	5	23	23	21.7	5.74	2.78E-04	1.87E-02
c2	MF	sequence-specific DNA binding transcription factor activity(GO:0003700)	49	1105	1105	4.4	3.87	3.17E-04	2.03E-02
c2	BP	regulation of cytokine secretion(GO:0050707)	10	102	102	9.8	4.55	3.50E-04	2.17E-02
c2	BP	negative regulation of RNA metabolic process(GO:0051253)	44	955	955	4.6	3.93	3.65E-04	2.25E-02
c2	MF	natural killer cell lectin-like receptor binding(GO:0046703)	4	14	14	28.6	6.08	3.81E-04	2.30E-02
c2	BP	regulation of wound healing(GO:0061041)	9	85	85	10.6	4.60	3.87E-04	2.32E-02
c2	BP	positive regulation of smooth muscle cell proliferation(GO:0048661)	7	54	54	13.0	4.76	5.02E-04	2.83E-02
c2	BP	regulation of natural killer cell chemotaxis(GO:2000501)	3	7	7	42.9	6.66	5.81E-04	3.18E-02
c2	BP	negative regulation of tumor necrosis factor biosynthetic process(GO:0042536)	3	7	7	42.9	6.66	5.81E-04	3.18E-02
c2	MF	calmodulin binding(GO:0005516)	13	172	172	7.6	4.06	6.34E-04	3.34E-02
c2	BP	regulation of phagocytosis(GO:0050764)	6	41	41	14.6	4.82	6.54E-04	3.42E-02
c2	BP	response to vitamin D(GO:0033280)	4	16	16	25.0	5.60	6.64E-04	3.44E-02
c2	CC	external side of plasma membrane(GO:0009897)	14	196	196	7.1	3.98	6.99E-04	3.54E-02
c2	BP	positive regulation of signaling(GO:0023056)	38	817	817	4.7	3.70	7.04E-04	3.54E-02
c2	BP	positive regulation of cell communication(GO:0010647)	38	817	817	4.7	3.70	7.04E-04	3.54E-02

c2	BP	negative regulation of homeostatic process(GO:0032845)	5	28	28	17.9	5.05	7.28E-04	3.59E-02
c2	BP	response to gamma radiation(GO:0010332)	6	42	42	14.3	4.73	7.47E-04	3.64E-02
c2	BP	positive regulation of leukocyte chemotaxis(GO:0002690)	7	58	58	12.1	4.51	7.78E-04	3.75E-02
c2	BP	monocyte differentiation(GO:0030224)	4	17	17	23.5	5.39	8.50E-04	4.01E-02
c2	BP	cytolysis(GO:0019835)	5	29	29	17.2	4.93	8.61E-04	4.03E-02
c2	CC	extracellular space(GO:0005615)	41	906	906	4.5	3.66	8.69E-04	4.04E-02
c2	BP	cellular response to interferon-alpha(GO:0035457)	3	8	8	37.5	6.17	9.12E-04	4.13E-02
c2	BP	regulation of endopeptidase activity(GO:0052548)	17	272	272	6.3	3.77	9.02E-04	4.13E-02
c2	BP	regulation of interleukin-1 beta production(GO:0032651)	6	44	44	13.6	4.57	9.61E-04	4.27E-02
c2	BP	negative regulation of cell proliferation(GO:0008285)	30	612	612	4.9	3.58	1.13E-03	4.82E-02
c2	BP	response to dsRNA(GO:0043331)	5	31	31	16.1	4.71	1.18E-03	4.97E-02
c2	BP	MAPKKK cascade(GO:0000165)	13	184	184	7.1	3.79	1.18E-03	4.97E-02

Table S3. 8-OHD Target Protein Prediction

Target	Common name	Target Class	Probability*	Known actives (3D/2D)
Xanthine dehydrogenase	XDH	Oxidoreductase	1.00	12 / 20
6-phosphofructo-2-kinase/fructose-2,6-bisphosphatase 3	PFKFB3	Enzyme	1.00	2 / 2
Arachidonate 12-lipoxygenase	ALOX12	Enzyme	0.90	9 / 11
Estrogen receptor alpha	ESR1	Nuclear receptor	0.37	78 / 71
Estrogen receptor beta	ESR2	Nuclear receptor	0.37	98 / 59
Arachidonate 15-lipoxygenase	ALOX15	Enzyme	0.35	6 / 8
Monoamine oxidase A	MAOA	Oxidoreductase	0.31	6 / 28
Adenosine A1 receptor (by homology)	ADORA1	Family A G protein-coupled receptor	0.31	6 / 16
Adenosine A2a receptor	ADORA2A	Family A G protein-coupled receptor	0.31	5 / 13
Estradiol 17-beta-dehydrogenase 1	HSD17B1	Enzyme	0.31	9 / 5
ATP-binding cassette sub-family G member 2	ABCG2	Primary active transporter	0.31	6 / 34
Thromboxane-A synthase	TBXAS1	Cytochrome P450	0.29	1 / 1
Epidermal growth factor receptor erbB1	EGFR	Kinase	0.29	9 / 8
Maltase-glucoamylase	MGAM	Hydrolase	0.29	1 / 1
Serotonin 2a (5-HT2a) receptor	HTR2A	Family A G protein-coupled receptor	0.29	1 / 1
Serotonin 2c (5-HT2c) receptor	HTR2C	Family A G protein-coupled receptor	0.29	1 / 2
Estrogen-related receptor alpha	ESRRA	Nuclear receptor	0.29	2 / 2

Estrogen-related receptor beta	ESRRB	Nuclear receptor	0.29	1 / 1
Carbonic anhydrase VII	CA7	Lyase	0.25	8 / 13
Carbonic anhydrase XII	CA12	Lyase	0.25	10 / 17
Carbonic anhydrase IV	CA4	Lyase	0.22	7 / 10
Tyrosinase (by homology)	TYR	Oxidoreductase	0.21	2 / 2
Macrophage migration inhibitory factor	MIF	Enzyme	0.21	1 / 7
Aldehyde dehydrogenase	ALDH2	Oxidoreductase	0.18	1 / 45
Cytochrome P450 19A1	CYP19A1	Cytochrome P450	0.17	6 / 24
Receptor-type tyrosine-protein phosphatase S	PTPRS	Phosphatase	0.15	7 / 7
Estradiol 17-beta-dehydrogenase 2	HSD17B2	Enzyme	0.15	9 / 4
NADPH oxidase 4	NOX4	Enzyme	0.13	6 / 7
P-glycoprotein 1	ABCB1	Primary active transporter	0.12	12 / 41
Interleukin-2	IL2	Secreted protein	0.12	0 / 4
Carbonyl reductase [NADPH] 1	CBR1	Enzyme	0.11	3 / 2
Acetylcholinesterase	ACHE	Hydrolase	0.10	4 / 32
Cyclooxygenase-1	PTGS1	Oxidoreductase	0.10	0 / 3
Norepinephrine transporter	SLC6A2	Electrochemical transporter	0.10	0 / 1
Serum paraoxonase/arylesterase 1	PON1	Enzyme	0.10	0 / 1
Carbonic anhydrase II	CA2	Lyase	0.10	8 / 22
Monoamine oxidase B	MAOB	Oxidoreductase	0.10	0 / 77
Alpha-synuclein	SNCA	Unclassified protein	0.10	2 / 0
Telomerase reverse transcriptase	TERT	Enzyme	0.10	9 / 10
Corticotropin releasing factor receptor 1	CRHR1	Family B G protein-coupled receptor	0.10	1 / 0

Insulin-like growth factor binding protein 3	IGFBP3	Secreted protein	0.10	3 / 0
Cyclooxygenase-2	PTGS2	Oxidoreductase	0.10	2 / 7
Steryl-sulfatase	STS	Enzyme	0.10	0 / 6
Toll-like receptor (TLR7/TLR9)	TLR9	Toll-like and Il-1 receptors	0.10	0 / 1
Carbonic anhydrase I	CA1	Lyase	0.10	4 / 21
Cyclin-dependent kinase 5/CDK5 activator 1	CDK5R1 CDK5	Kinase	0.10	6 / 6
Arachidonate 5-lipoxygenase	ALOX5	Oxidoreductase	0.10	5 / 26
Induced myeloid leukemia cell differentiation protein Mcl-1	MCL1	Other cytosolic protein	0.10	5 / 4
Aldose reductase (by homology)	AKR1B1	Enzyme	0.10	17 / 51
Tyrosine-protein kinase receptor FLT3	FLT3	Kinase	0.10	6 / 6
Multidrug resistance-associated protein 1	ABCC1	Primary active transporter	0.10	7 / 10
Cytochrome P450 1B1	CYP1B1	Cytochrome P450	0.10	12 / 22
Cyclin-dependent kinase 1/cyclin B	CCNB3 CDK1 CCNB1 CCNB2	Other cytosolic protein	0.10	4 / 4
Cyclin-dependent kinase 6	CDK6	Kinase	0.10	3 / 3
Tyrosine-protein kinase SYK	SYK	Kinase	0.10	3 / 3
Glycogen synthase kinase-3 beta	GSK3B	Kinase	0.10	3 / 7
Transthyretin	TTR	Secreted protein	0.10	2 / 2
Casein kinase II alpha	CSNK2A1	Kinase	0.10	4 / 3
Cystic fibrosis transmembrane conductance regulator	CFTR	Other ion channel	0.10	1 / 1
Aldo-keto reductase family 1 member B10	AKR1B10	Enzyme	0.10	2 / 3
Tankyrase-2	TNKS2	Enzyme	0.10	4 / 7

Tankyrase-1	TNKS	Enzyme	0.10	4 / 10
Protein farnesyltransferase	FNTA FNTB	Enzyme	0.10	1 / 0
Serine/threonine-protein kinase PIM1	PIM1	Kinase	0.10	8 / 5
Serine/threonine-protein kinase Aurora-B	AURKB	Kinase	0.10	3 / 4
Insulin-like growth factor binding protein 6	IGFBP6	Secreted protein	0.10	2 / 0
Insulin-like growth factor binding protein 4	IGFBP4	Secreted protein	0.10	2 / 0
Insulin-like growth factor binding protein 5	IGFBP5	Secreted protein	0.10	2 / 0
Insulin-like growth factor binding protein 2	IGFBP2	Secreted protein	0.10	2 / 0
Insulin-like growth factor binding protein 1	IGFBP1	Secreted protein	0.10	2 / 0
Insulin-like growth factor I receptor	IGF1R	Kinase	0.10	3 / 3

Table S4. 8-OHD affects Ara-C-metabolizing gene expression

Gene symbol	Description	log_2FC	*P.* value
CDA	cytidine deaminase	-0.680175	0.0027
DCK	deoxycytidine kinase	0.606318	0.0073
RRM2	ribonucleotide reductase M2	1.44291	0.0001

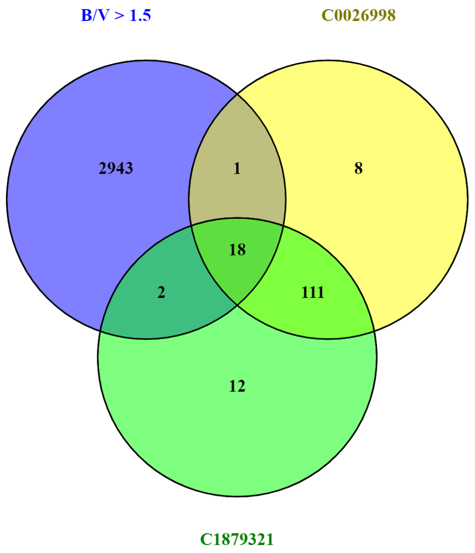

CCND2
MYC
NTRK3
POU4F1
NPM1
SYNGR1
CD9
FLT3
TERT
WT1
BCL2
NSD1
MN1
HSPB1
ZBTB7A
TCEA2
ENAH
ERG

Figure S1. Venn diagram of 8-OHD-downregulated DEGs (B/V>1.5 and $p < 0.05$) and gene sets of DisGeNET CUI: C0026998 and CUI: C1879321. The 18 genes in the intersection of three sets were listed at right side.

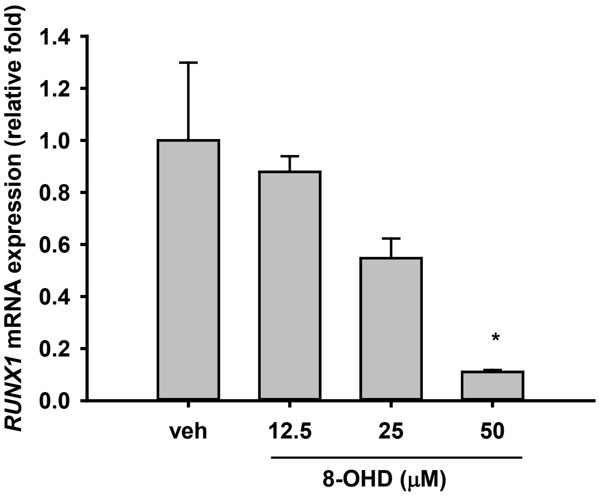

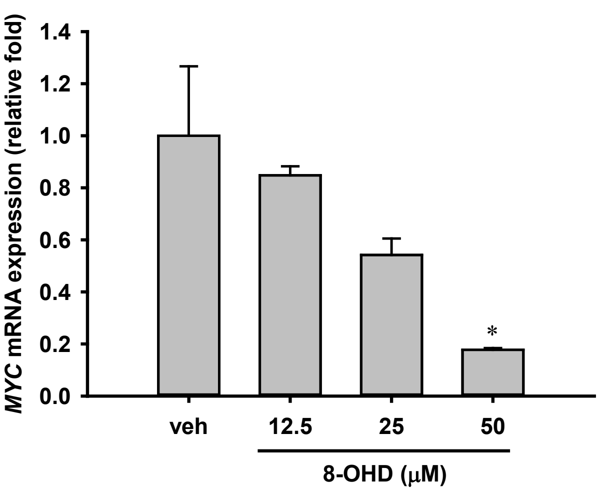

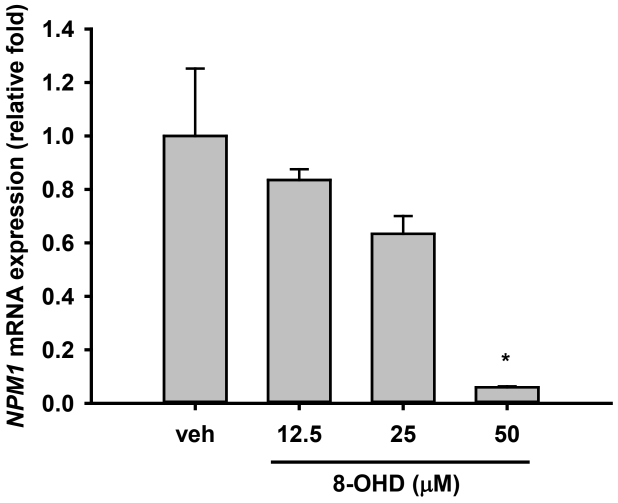

(c)

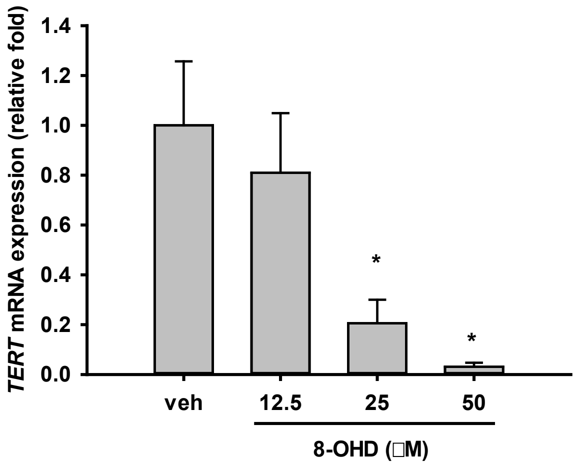

(d)

Figure S2. GSEA result indicates that the gene set "Hallmark Myc Targets v.1" is enriched with 8-OHD-downregulated DEGs. The barcode plot indicates the position of the genes in each gene set. The horizontal bar in graded color from red to blue indicates up- and down-regulated by 8-OHD. The vertical axis in the lower plot indicates Ranked List Metri

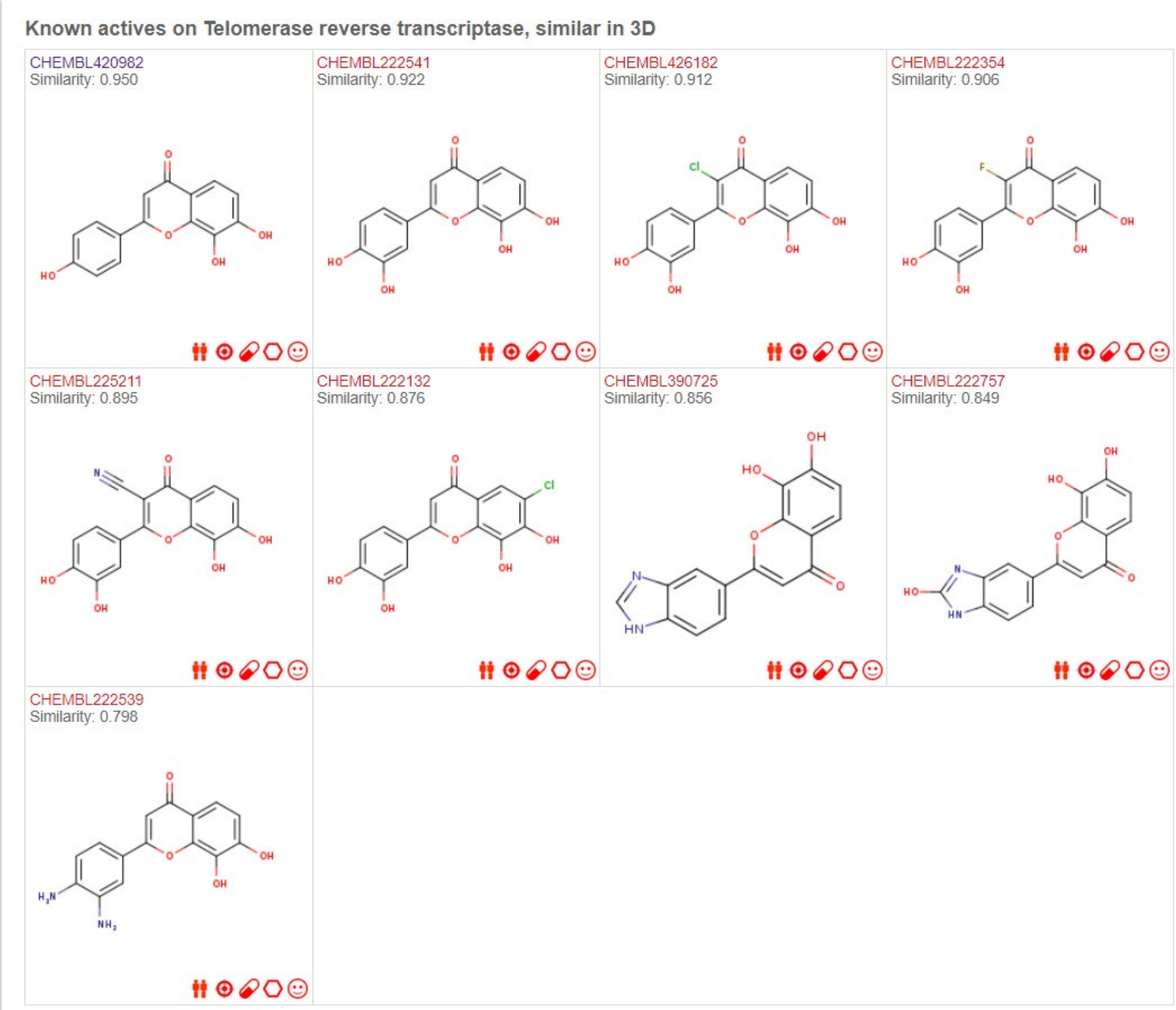
Known actives on Telomerase reverse transcriptase, similar in 3D
CHEMBL420982
Similarity: 0.950
CHEMBL222541
Similarity: 0.922
CHEMBL426182
Similarity: 0.912
CHEMBL222354
Similarity: 0.906
CHEMBL225211
Similarity: 0.895
CHEMBL222132
Similarity: 0.876
CHEMBL390725
Similarity: 0.856
CHEMBL222757
Similarity: 0.849
CHEMBL222539
Similarity: 0.798

(a)

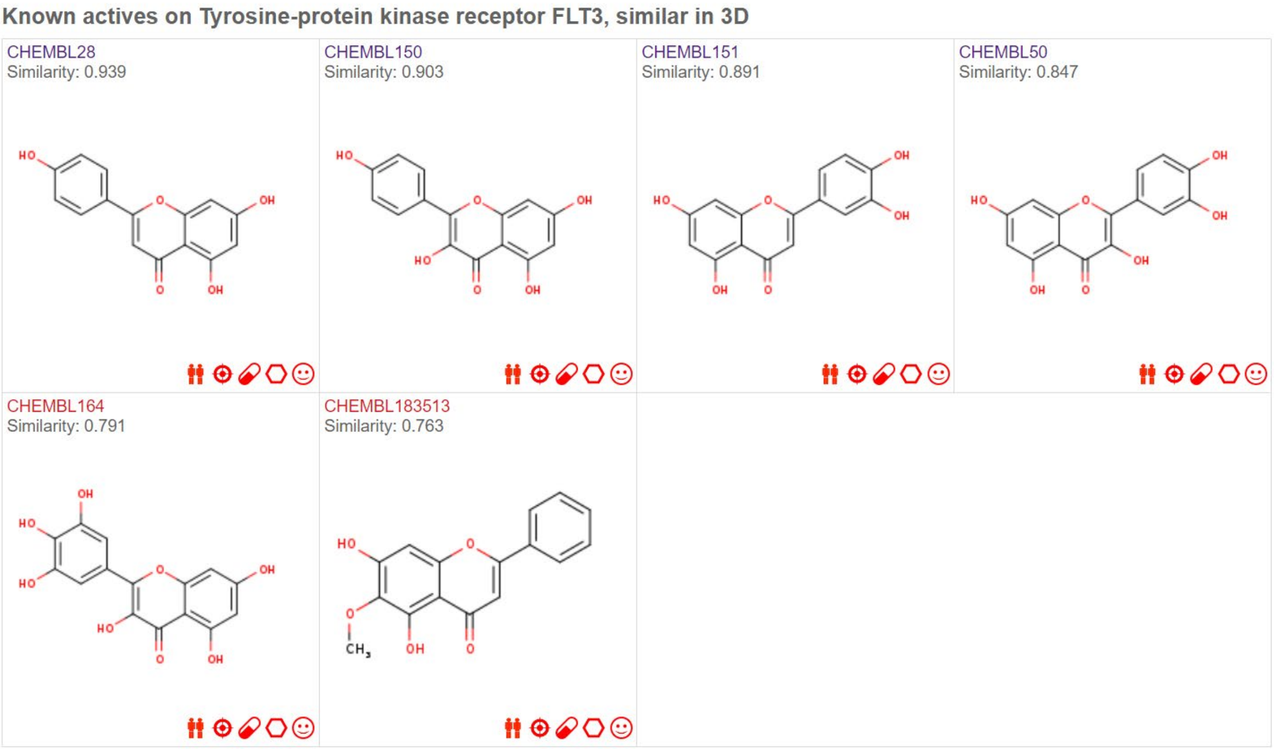

(b)

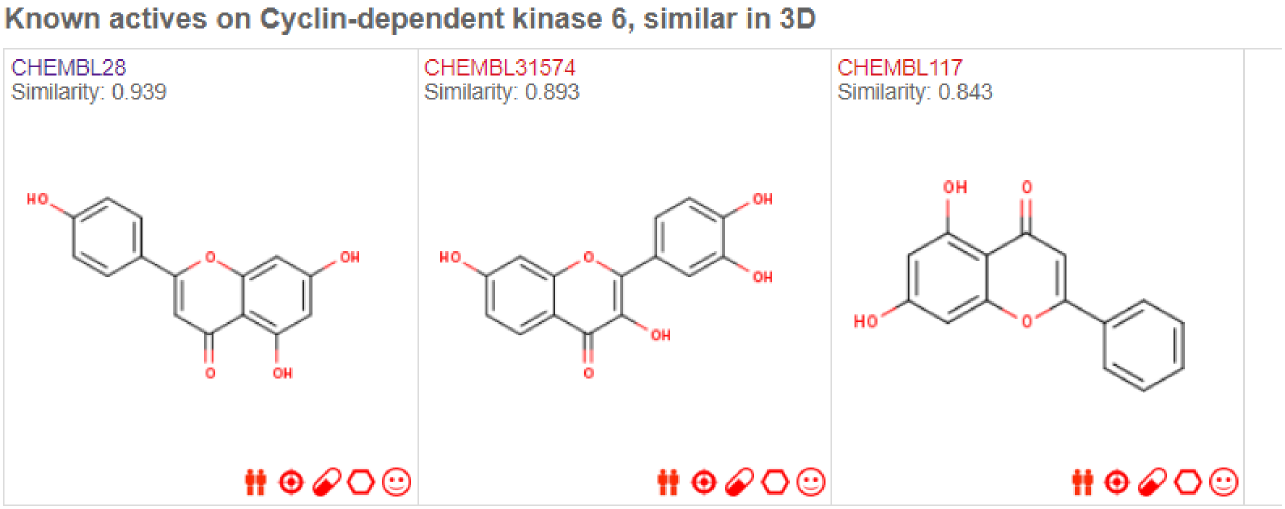

(c)

Figure S3. Effects of 8-OHD on the expression of *RUNX1, MYC, NPM1* and *TERT* in THP-1 cells. RNA was prepared from THP-1 cells which were treated with 50 μM 8-OHD for 24 h. The gene expression was measured by RT-qPCR as described in Materials and Methods.

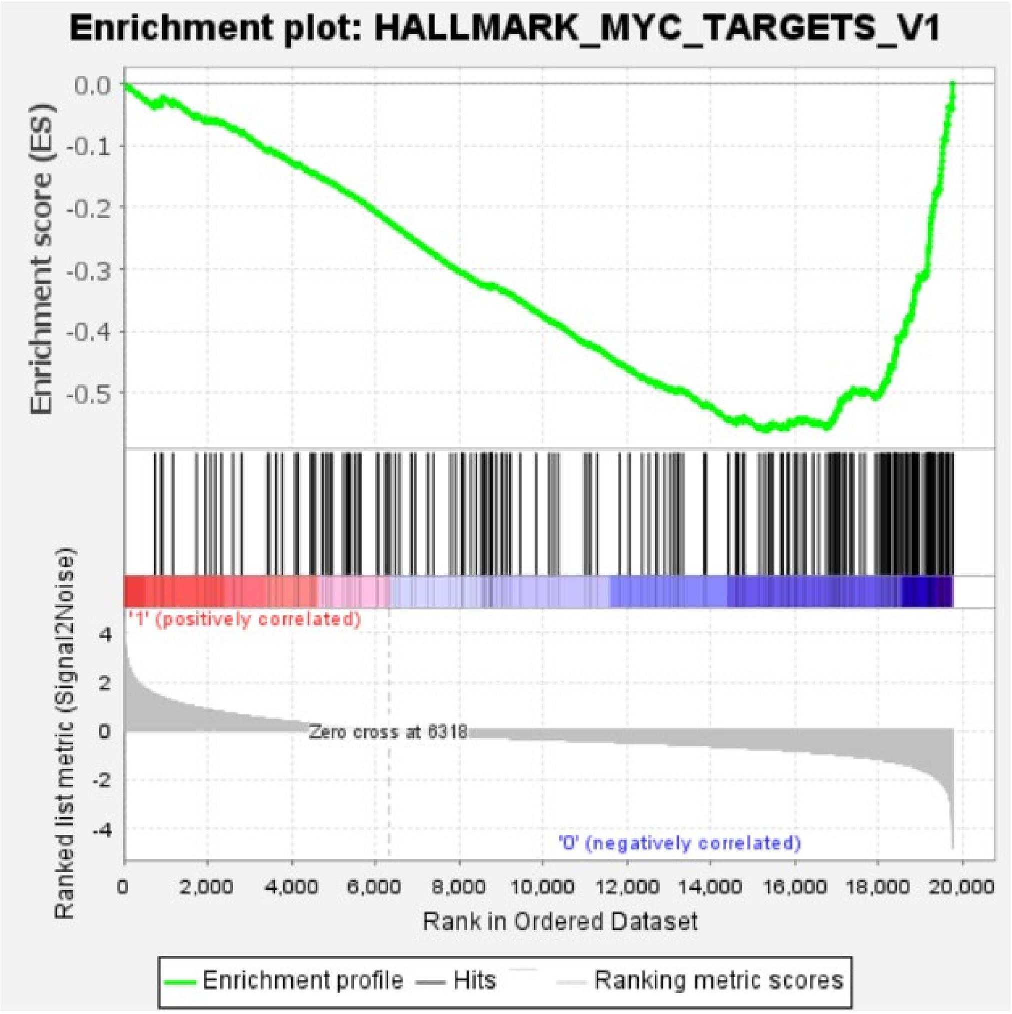

Figure S4. Known actives on TERT, FLT3 and CDK6 that are highly similar to 8-OHD according to 3D measures.
